# Synthetic Fractal Modelling of Heterogeneous and Anisotropic Reservoirs for Use in Simulation Studies: Implications on Their Hydrocarbon Recovery Prediction

**DOI:** 10.1007/s11242-016-0770-3

**Published:** 2016-10-01

**Authors:** Saud Al-Zainaldin, Paul W. J. Glover, Piroska Lorinczi

**Affiliations:** School of Earth and Environment, University of Leeds, Leeds, LS2 9JT UK

**Keywords:** Heterogeneous reservoirs, Anisotropy, Fractal dimension, Reservoir simulation, Reservoir modelling, Poroperm

## Abstract

Optimising production from heterogeneous and anisotropic reservoirs challenges the modern hydrocarbon industry because such reservoirs exhibit extreme inter-well variability making them very hard to model. Reasonable reservoir models can be obtained using modern geostatistical techniques, but all of them rely on significant variability in the reservoir only occurring at a scale at or larger than the inter-well spacing. In this paper we take a different, generic approach. We have developed a method for constructing realistic synthetic heterogeneous and anisotropic reservoirs which can be made to represent the reservoir under test. The main physical properties of these synthetic reservoirs are distributed fractally. The models are fully controlled and reproducible and can be extended to model multiple facies reservoir types. This paper shows how the models can be constructed and how they have been tested. Reservoir simulation results of a number of generated 3-D heterogeneous and anisotropic models show that heterogeneity, in terms of only the geometric distribution of reservoir properties, has a little effect on oil production from high and moderate quality reservoirs. However, if the effect of heterogeneity on capillary pressure is taken into account, the effect becomes striking, where varying the heterogeneity of reservoirs properties can lead to a 70 % change in the predicted oil production rate and a significant early shift of water breakthrough time. Hence, it is the heterogeneity consequences that are really substantial if not taken into account. These are very significant uncertainties for a hydrocarbon company if the heterogeneity of their reservoir is not well defined.

## Introduction

Reservoir production performance and ultimate hydrocarbon recovery are controlled by both the reservoir fluid transport properties (e.g. porosity and permeability) and pore geometry (Mandal et al. 2006). Reservoir performance is also controlled by the spatial variation of these reservoir properties (i.e. heterogeneity) (Li 2010; Perez and Chopra 1997; Shen et al. 1998). Optimising the production from heterogeneous and anisotropic reservoirs has challenged the oil industry for a long time. This stems from the fact that reservoir modelling and simulation of such reservoirs is difficult as their inter-well volumes are extremely hard to model in a reliable fashion. As a result, understanding the non-uniform distribution of reservoir properties and how they distribute themselves in the porous media is vital in studying the oil recovery (Hewett 2001). To describe a reservoir effectively, it is important to use the appropriate approach to quantitatively characterise its heterogeneity (Li 2010).

Modelling the spatial variation of reservoir properties (e.g. porosity and permeability) at laboratory scale, at which they are measured, is important to account for the effect of their heterogeneous distribution (Hewett 1993). Unfortunately, such data are only available at some locations, usually wells, and a complete reservoir description at this scale cannot be obtained. Recently developed statistical techniques can go some way towards helping to construct a reasonable reservoir model, but all of them rely on significant variability in the reservoir only occurring at a scale at or larger than the inter-well spacing (Al Qassab et al. 2000; Al-Ali and Al-Qassab 2000; Alqassab and Heine 1998). Because such data are impossible to obtain for the whole reservoir, it was decided to construct a synthetic property distribution mimicking real reservoirs and representing the scale necessary for reservoir simulation and representing the macroscopic laboratory measurements at the same time. These synthetic models can then be used to study the generic influence of heterogeneity on hydrocarbon production.

Several approaches were developed to characterise the heterogeneity of objects in nature. It has been shown that sandstones have fractal geometric pore spaces (Katz and Thompson 1985; Krohn and Thompson 1986; Krohn 1988a, b; Yu and Li 2001). This implies that a fractal approach can be implemented to characterise the spatial heterogeneity of reservoir properties (Perez and Chopra 1997). Fractals are powerful since they have the ability to describe the broad range of variability which exists in reservoir properties such as porosity and permeability (Perez and Chopra 1997).

In this paper, we take a different, generic approach. A method for constructing synthetic, but realistic, heterogeneous and anisotropic reservoirs, which can be made to represent the reservoir under test, was developed using the fractal approach. The tested and produced reservoir models have fractally distributed porosity, grain size, cementation exponent, and permeability, mimicking the fractal behaviour observed in real reservoirs. The fractal approach was implemented in order to study quantitatively the effect of heterogeneity and anisotropy of reservoirs on their production performance. The degree of heterogeneity can be modelled by varying the fractal dimension. Moreover, anisotropy can be implemented in the algorithm using a simple factor. It should be noted that this methodology is not aimed at modelling a specific reservoir, but rather constructing synthetic ones that can have different scenarios of heterogeneity and anisotropy degrees. These synthetic models can then be used to study the generic influence of heterogeneity and anisotropy on hydrocarbon production.

This paper shows how to generate and test fractal models that can be used as models for reservoir properties with variable degrees of spatial correlation (i.e. heterogeneity). We are interested in addressing how the different realisations of reservoir properties affect the reservoir performance prediction through time. This is achieved by synthesising fractal volumes using fractional Brownian motion *“fBm”* algorithm which represent reservoir properties that are distributed fractally within the reservoir. After that, a dynamic simulation has been conducted to predict the reservoir performance with several heterogeneity and anisotropy scenarios in order to study their effect on oil production.

## Fractal Models of Reservoir Parameters

### Fractal Pore Space

Reservoir properties are measured indirectly, by geophysical surveys, or directly but at certain locations by drilling wells. Due to the Earth’s complexity, it is considered to be a big challenge to characterise and model inter-well reservoir properties. The complexity stems from the fact that it is difficult to infer the location, distribution, and structure of rock types (i.e. grain size distribution, porosity, permeability) away from the wells where they were measured (Dimri et al. 2012).

The fractal concept of Mandelbrot (1977) has found various applications in various geoscience sectors. This is due to the fact that many physical systems in nature produce a variation of properties that can be described via fractal statistics (Lozada-Zumaeta et al. 2012). Several studies have been carried out in the past decades relating the fractal theory to the distribution of reservoir properties such as porosity and permeability. Turcotte (1997) modelled the sedimentation process by the devil’s staircase, which is an exact fractal, and showed that the rate of sedimentation can be related to the time interval of deposition occurrence by a fractal power law relationship. Moreover, the soil particles can be represented as a fragmented porous medium (Rieu and Sposito 1991). Turcotte (1997) showed that the frequency size distribution of fragments is fractal, with the number of fragments depending on their linear dimension through a fractal power law relationship. Since the formation of porosity is closely linked to both sedimentation processes and to fragmentation, both of which show fractal behaviour, it makes sense that porosity would be expected to follow a fractal behaviour too. In other words, if the grain size distribution is fractal, then the pore size would also be expected to be distributed fractally (Turcotte 1997; Tyler and Wheatcraft 1990).

Laboratory measurements have confirmed that the porosity of sandstones is indeed fractal, exhibiting a non-integer power law scaling behaviour (Katz and Thompson 1985; Krohn and Thompson 1986; Krohn 1988a, b; Thompson 1991; Thompson et al. 1987). Katz and Thompson (1985) showed that a set of sandstone samples had fractal geometric pore spaces which were self-similar over three to four orders of magnitude in length in the range 10 Å to $100\,\upmu \hbox {m}$. Additionally, Krohn (1988a) reported a study on the microstructure of sandstone, shale and carbonate samples, by statistically analysing the structural features on their fracture surfaces. He argued that fractal behaviour can be confirmed if the number of features as a function of feature size on the pore–rock interface shows a power law (self-similar) behaviour, which was the case over a very large scale range, representing the limits of self-similarity.

### Heterogeneity Characterisation by Fractal Dimension

Studies on sandstone samples and cores reveal that homogenous samples with good sorting and little clay content have small fractal dimensions, while samples having bad sorting and bad roundness with secondary mineralisation or more clay content have high fractal dimension (Hewett 1986, 1993; Li 2010; Mandal et al. 2006; Shen et al. 1998). These studies concluded that the smaller the fractal dimension, the more homogeneous the pore structure and the better the reservoir storage capacity. On the other hand, the higher the fractal dimension, the more heterogeneous the pore structure and the less the reservoir capacity. Note that the heterogeneity defined by the fractal dimension is the microscopic-scale heterogeneity. However, this occurs over large range of scale. Based on rock sample measurements, Li and Xie (2011) argued that rock samples with high fractal dimensions were also heterogeneous at macroscopic scale.

The great advantage of these studies is that the heterogeneity of rock samples can be characterised quantitatively by the fractal dimension. This allows the construction of reservoir property distribution models with different scenarios of the degree of heterogeneity, modelled by fractal dimension, and their subsequent use as inputs to reservoir simulation in order to study the effect of property heterogeneity on reservoir performance prediction.

### Synthetic Fractal Reservoirs Property Distribution

The statistics of sediment distribution is clearly controlled by the natural processes which created them. Since these processes have been proven to behave fractally, it is expected that the distribution of sediments will show fractal behaviour too (Lozada-Zumaeta et al. 2012). Consequently, a desired spatial reservoir petrophysical property distribution can possibly be modelled with fractal distribution, with the desired power law behaviour, over a finite range of scales defining the self-similar validity range (Hewett 1986, 1993).

A good model to describe the spatial statistics of reservoir property distribution would be random fractals that have self-similar statistics. This can be achieved by synthesising random fractal distribution and then using it to model the property distribution of properties showing fractal behaviour, such as porosity and grain size distributions (Hewett 1986). In this paper, it is not intended to infer the heterogeneity between wells of a specific reservoir, but rather to assume that a synthesised fractal distribution reveals the available geologic information of a reservoir (i.e. similar to have a well everywhere in a real reservoir). Then, to try to answer the questions “to what extent do the heterogeneity and degree of anisotropy, if not accounted for, affect our ability to optimise heterogeneous and anisotropic reservoirs? Can we analyse, quantitatively, the relationship between the degree of heterogeneity and anisotropy and our ability to predict the reservoir performance?”

As far as we are aware, the presented framework for generating synthetic reservoirs using 3-D fractal modelling has not been used before for such studies. All previous applications to the fractal approach were for specific reservoirs, whereas our methodology is general and not specific to a certain reservoir. Examples of such specific studies were done by Shen et al. (1998), Perez and Chopra (1997), and Hewett and Behrens (1990). While these studies aim to study the effect of heterogeneity on a certain reservoir, our intention is to study this effect in a generic manner.

## Modelling Methodology

The construction of geological reservoir models was carried out using the following five steps.

- **I.**    **3-D fractal volume (fractional Brownian motion,** *fBm* **) generation**
  - Generating independent fractal volumes which share some physical features using the fBm algorithm.
  - Renormalising the resultant volumes between zero and unity.
- **II.**    **Fractal property modelling**
  - Rescaling these fractal volumes to the desired porosity, grain size, and cementation exponent volumes.
- **III.**    **Permeability modelling**
  - Use porosity, grain size, and cementation factor volumes to calculate the absolute permeability using the RGPZ equation.
- **IV.**    **Fluid saturations modelling**
  - Calculate fluid saturations using the Brooks–Corey model, which relates the capillary and capillary entry pressures to water saturation.
- **V.**    **Relative permeability modelling**
  - Calculate the relative permeability curves using Brooks–Corey–Mualem method.

The main advantage of this methodology is that it enables us to generate synthetic reservoir models with different scenarios of heterogeneity and anisotropy for reservoir performance prediction without the need of any hard data of any given reservoir.

### Generating 3-D Fractal Volumes

Fractional Brownian motion (*fBm*) (Mandelbrot and Van Ness 1968; Saupe 1988) has been recognised as one of the most powerful mathematical models to simulate random fractals which enabled the modelling of complex objects found in nature such as clouds and mountains (Voss 1988). Several algorithms are available for simulating finite *fBm* objects including (i) the midpoint displacement method, (ii) the successive random addition method, and (iii) the spectral synthesis method (Saupe 1988). The spectral synthesis method has been used previously to produce synthetic rough fractures in porous media by Isakov et al. (2001), Ogilvie et al. (2002), Ogilvie et al. (2003), and Ogilvie et al. (2006), who based their work on that of Glover et al. (1997, (1998), and Brown (1995). The developments in this study allow such an approach to be applied to the petrophysical characterisation and modelling of hydrocarbon reservoirs.

The spectral synthesis method, which is also known as the Fourier filtering method, has been implemented based on the construction of a random function with the desired spectral density such that it follows a non-integer power law scaling behaviour. According to Saupe (1988), a random process $X( {t_1,t_2 ,\ldots ,t_{n}})$ with a spectral density $S( {f_1 ,f_2 ,\ldots ,f_E } )$ is fractional Brownian motion (*fBm*) if

1 $$\begin{aligned} S\left( {f_1 ,f_2 ,\ldots ,f_E } \right) \propto \left( {\sqrt{\mathop \sum \limits _{i=1}^{E} f_{i}^{2}}} \right) ^{-\beta }, \end{aligned}$$

where the spectral exponent $\beta$ is related to the Hurst exponent *H* ($0 \le H \le 1$) as

2 $$\begin{aligned} \beta =2H+E, \end{aligned}$$

and *H* is related to the fractal dimension $\mathcal {D}$ through

3 $$\begin{aligned} \mathcal {D}=n+1-H, \end{aligned}$$

where *n* is the simple Euclidean dimension (1 for 1-D curves, 2 for 2-D surfaces, and 3 for 3-D volumes). The fractal dimension can only be between 1 and 2 in the 1-D case, 2 and 3 in the 2-D case, while it ranges between 3 and 4 in the 3-D case.

It can be noted that *fBm* fractal dimension exceeds the topological dimension by one. This is due to *fBm* being self-affine rather than self-similar. For a self-affine object, the fractal dimension is not as easily defined as with self-similarity. Self-affinity is an important property of *fBm* and means that it is invariant statistically if different coordinates are rescaled by different amounts (Mandelbrot and Van Ness 1968). For a self-affine time series to repeat itself “statistically”, both axes (e.g. *t* and *V*) should be magnified by different amounts. If the time *t* is rescaled by a factor *x*, then *V* must be rescaled by a factor $x^{H}$, which is what we call “self-affinity” (Voss 1988). Thus, the similarity dimension implicitly fixes a scaling between the “otherwise independent” coordinates (Family and Vicsek 1991).

However, fractals typically reduce their dimension by one if intersected with a plane. This is a property of Euclidean objects as well. If a 2-D plane intersects a 1-D line, they will intersect in 0-D points. On the other hand, the intersection of a 3-D cube, or sphere, with a plane will result in a 2-D intersection plane. Similarly, when a self-affine fractal curve, with a fractal dimension between 1 and 2, intersects a straight line, the result is a set of points, called zeroset. Although the original object is self-affine, this set of points is self-similar, not self-affine. The importance of this concept is that a self-similar fractal can be generated by reducing a self-affine fractal. Therefore, this set of points, or zeroset, will have a fractal dimension of $\mathcal {D}_0 =1-H$ and the fractal dimension of the self-affine *fBm* would be $\mathcal {D}=\mathcal {D}_0 +1$. Using this concept, the relationship of the fractal dimension of a self-affine fBm curve to the scaling parameter *H* would be $\mathcal {D}=2-H$. In general, the fractal dimension of a statistically self-affine *fBm* in *n* Euclidean dimension is related to *H* by Eq. 3. This implies that the fractal dimension of a 2-D fractal *fBm* surface is $\mathcal {D}=3-H$, while a 3-D fractal *fBm* would have a fractal dimension of $\mathcal {D}=4-H$. Their zerosets, however, form a statistically self-similar fractal with fractal dimension $\mathcal {D}_0 =2-H$ and $\mathcal {D}_0 =3-H$, respectively (Voss 1988). *FBm* fractal dimension works as a correlation parameter, where low value means a high correlation between the nearby cells.

Equations 1, 2, and 3 imply that a three-dimensional *fBm* has a spectral density depending on three frequencies corresponding to *x*, *y*, and *z* directions

4 $$\begin{aligned} S( {f_1 ,f_2 ,f_3 } )\alpha \frac{1}{\left( {f_{1}^{2} +f_{2}^{2} +f_{3}^{2} } \right) ^{\frac{\beta }{2}}}\quad \hbox {with}\quad \beta =2H+3. \end{aligned}$$

To synthesise a 3-D fractional Brownian motion with the Fourier filtering method, the discrete Fourier transform is considered. It can be written as

5 $$\begin{aligned} X( {x,y,z} )=\mathop \sum \limits _{k=0}^{N-1} \mathop \sum \limits _{l=0}^{N-1} \mathop \sum \limits _{w=0}^{N-1} a_{klw} \mathrm{e}^{2\pi i\left( {k_x +l_y +w_z } \right) }, \end{aligned}$$

where $a_{klw}$ is the coefficient for three-dimensional fast Fourier transform (FFT). This expression contains information about the amplitude spectrum $| {a_{klw} } |$ and phase spectrum $( {2\pi ( {k_x +l_y +w_z } )} )$ of the function *X*( *x*, *y*, *z* ). It can be rewritten as

6 $$\begin{aligned} X( {x,y,z} )= & {} \left| {a_{klw} } \right| \mathop \sum \limits _{k=0}^{N-1} \mathop \sum \limits _{l=0}^{N-1} \mathop \sum \limits _{w=0}^{N-1} \left[ \left[ {\cos \left( {2\pi \left( {k_x +l_y +w_z } \right) } \right) } \right] \right. \nonumber \\&\left. +i\left[ {\sin \left( {2\pi \left( {k_x +l_y +w_z } \right) } \right) } \right] \right] . \end{aligned}$$

In order to obtain a fractal behaviour, conditions in Eq. 4 are to be imposed such that

7 $$\begin{aligned} E\left( {\left| {a_{klw} } \right| ^{2}} \right) \alpha \frac{1}{\left( {k^{2}+l^{2}+w^{2}} \right) ^{\frac{2H+3}{2}}}, \end{aligned}$$

where *k*, *l*, and *w* are the directional frequency components. As a result, the Fourier coefficients are calculated as

8 $$\begin{aligned} a_{klw} =\frac{1}{\left( {k^{2}+l^{2}+w^{2}} \right) ^{\frac{2H+3}{4}}}. \end{aligned}$$

The algorithm is simply implemented by constructing a 3-D matrix with the desired dimensional size, which represents the Fourier transform coefficients of Eq. 5. The matrix is populated with random phases drawn from a Gaussian “normal” distribution^1^ with zero mean and a standard deviation of unity, where the phase at each location *X*( *x*, *y*, *z* ) is $\varphi _{kl} =2\pi \cdot rand_{klw}$ and the parameter $rand_{klw}$ is derived from a high-quality random number generator. At the same time, at each of the coordinates *x*, *y*, and *z*, the amplitude spectrum coefficients $a_{klw}$ are populated such that they conform to the power law in Eq. 8. Subsequently, both the real and imaginary parts of the Fourier complex coefficients matrix

9 $$\begin{aligned} X\left( {x,y,z} \right) =\left[ {a_{klw} \cdot cos \,\varphi _{klw} } \right] +i\left[ {a_{klw} \cdot sin\, \varphi _{klw} } \right] \end{aligned}$$

can be calculated at each matrix location, where $a_{klw}$ and $\varphi _{klw}$ are the amplitude and phase spectrum of Fourier transform. Finally, an inverse fast Fourier transform (IFFT) is performed to obtain the desired three-dimensional fractal volume. For the IFFT to return real values, the original matrix has been restricted to be symmetric. Conjugate symmetry is applied so that all complex terms of *X*( *x*, *y*, *z* ) will cancel each other after the IFFT. The resulting 3-D volume values are spatially correlated by the imposed fractal dimension. The higher the fractal dimension, the rougher the geometric distribution will be. On the other hand, the lower the fractal dimension, the smoother the geometric distribution will be. Equation 1 implies that log(frequency) is linearly related to log(magnitude) and that the gradient of the power law allows the spectral exponent $\beta$ to be calculated, from which the fractal dimension $\mathcal {D}$ of the volume can be derived. If the behaviour is not linear in log–log space, the object does not have fractal behaviour (Brown 1995; Russ 1994). Anisotropy can be introduced into the algorithm by modifying the Fourier coefficients of the spectral density function. In practice, this simply means we need to multiply each frequency component that corresponds to a certain spatial direction by a constant factor, which we call the anisotropy factor $\chi$ (Brown 1995). This implies that the modified equation will replace Eq. 8 in the algorithm. The new equation is

10 $$\begin{aligned} a_{klw} =\frac{1}{\left( {\left( {\frac{k}{\chi _{x}}} \right) ^{2}+\left( {\frac{l}{\chi _{y}}} \right) ^{2}+\left( {\frac{w}{\chi _{z}}} \right) ^{2}} \right) ^{\frac{2H+3}{4}}}. \end{aligned}$$

In this paper, the anisotropy is allowed to vary only in the *z* direction, while lateral anisotropies were held at unity. It is possible to introduce anisotropies into the lateral directions, and when implemented they behave perfectly symmetrically. Consequently, in this paper, the value of the anisotropy factor $\chi$ should be taken to represent the anisotropy in the *z* direction only and varies between 0 and infinity, with $\chi =1$ representing isotropic behaviour (i.e. no anisotropy in any direction).

The fractal volume generation process relies on defining random phases which can be seeded. Each seed of a random number generator produces a different set of random numbers and hence a different fractal volume, and the use of the same seed always produces the same volume. Hence, the random number generator can be treated as a texture classification generator. In this way, any generated fractal volume can be reproduced at any time with any combination of fractal dimensions and anisotropies. This gives us total control over the resulting volumes. Alternatively, we may keep the same fractal dimension and anisotropy factor and create many volumes with different seeds “or texture classification numbers”. This ultimately allows us to explore the range of the resulting oil and gas production rates from different implementations that share the same physical characteristics. In this sense, the model that we have created is fully determinative and not stochastic at all, as stochastic implies reliance on a random process to provide a random result. Some people might argue that the use of a random number to generate the *fBm* fractal distribution makes the model stochastic, but a truly stochastic model is one that cannot be repeated, whereas in our case the models are repeatable. Since the model is fully determined, uncertainty quantification analysis is not needed—it is what it is.

**Fig. 1:**
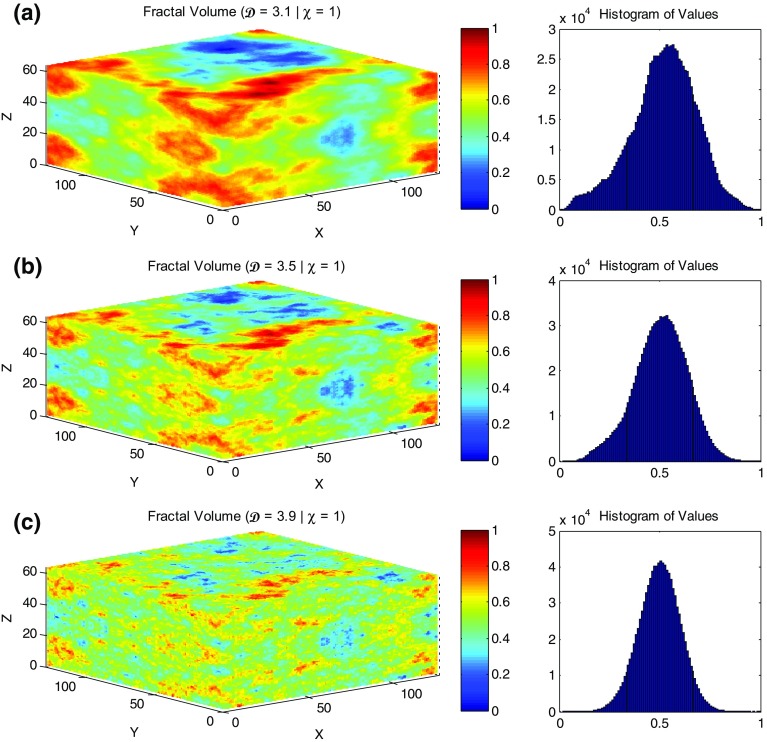
Isotropic fractal volumes with their resulting distribution histograms with **a** $\mathcal {D}=3.1$, $\chi =1$; **b** $\mathcal {D}=3.5$, $\chi =1$; **c** $\mathcal {D}=3.9$, $\chi =1$. The *x*, *y*, and *z* axes represent the number of blocks at each direction which are ($128 \times 128 \times 64$; 1,048,576 voxels) in these particular examples

Advantages of the spectral synthesis method include its simplicity to understand and to implement and code as well as its efficiency generating fractal objects (curves, surfaces, or volumes). However, because of the periodical nature of Fourier transforms, sometimes we end up computing twice as many points as needed. In this case, we need to discard a part of the resulting object that is not needed (Saupe 1988).

Figure 1 shows a typical example of an isotropic fractal realisation (anisotropy factor $\chi =1$) with three different fractal dimensions ($\mathcal {D}=3.1$, 3.5, and 3.9, respectively) together with their value magnitude histograms. The effect of increasing the fractal dimension is clear, leading to increasing variability of the fractal volumes. It should be noted that the bottom half of the volumes are mirror images of their top halves. This is an artefact of the fractal volume generating method and does not pose a problem because one of the halves can be disregarded. This process does not change the overall fractal dimension or affect the statistical normal distributions to which all the voxels of the model contribute.

**Fig. 2:**
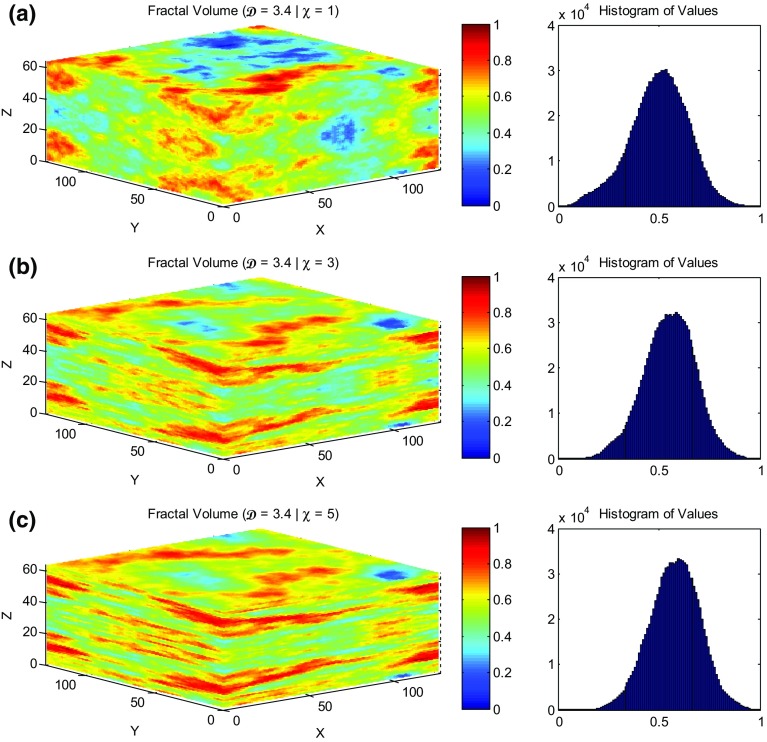
Anisotropic fractal volumes with their resulting distribution histograms with **a** $\mathcal {D}=3.4$, $\chi =1$; **b** $\mathcal {D}=3.4$, $\chi =3$; **c** $\mathcal {D}=3.4$, $\chi =5$. The *x*, *y*, and *z* axes represent the number of blocks at each direction which are ($128 \times 128 \times 64$; 1,048,576 voxels) in these particular examples

Figure 2 shows three typical fractal realisations together with their distributions, but this time with changing the vertical anisotropy factor. The fractal dimension is fixed in all the three ($\mathcal {D}=3.4$), while the anisotropy factors are $\chi =1$, 3, and 5, respectively. The operation of the anisotropy factor in the second and third volumes is very obvious acting as a layering introducer to the model.

### Testing the Models

The reservoir model building process has been tested at every stage. The initial volumes were tested for fractal behaviour and to ensure that they have their intended fractal dimension. This analysis was performed using a MATLAB code developed by Zhang (2005), is obtained from the MATLAB Central File Exchange, and is independent of any of our procedures. This MATLAB code measures the fractal dimension using the spectral density function and the spectral exponent $\beta$. Taking the natural logarithm of both sides of the three-dimensional form of Eq. 1 results in

11 $$\begin{aligned} \log S\left( {f_1 ,f_2 ,f_3 } \right) =-\beta \log \left( {\sqrt{f_1^2 +f_2^2 +f_3^2 }} \right) , \end{aligned}$$

where the exponent $\beta$ is related to the fractal dimension $\mathcal {D}$ through Eqs. 2 and 3 by

12 $$\begin{aligned} \mathcal {D}=\frac{11-\beta }{2}. \end{aligned}$$

Equation 11 shows that the frequency is linearly related to the magnitude on a log–log scale, which the validation code measures and plots. If the double logarithmic plot of the three components as a function of frequency is linear, the object is fractal. The gradient of the line can be used to calculate the spectral exponent $\beta$, from which the fractal dimension may be calculated using Eq. 12 (Russ 1994). If the relationship is not linear on a double logarithmic plot, the object lacks fractal behaviour. The static statistical distributions of all volumes were also tested to ensure that they were normally distributed with a mean and standard deviation corresponding to the imposed values after normalisation.

**Fig. 3:**
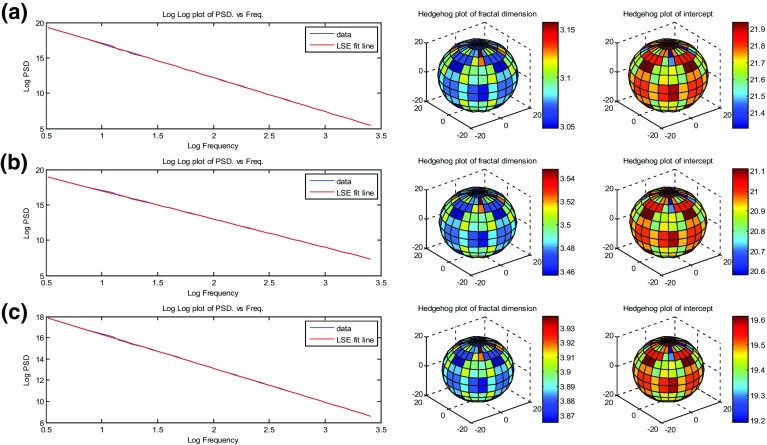
Fractal behaviour tests performed on fractal volumes shown in Fig. 1, where **a** $\mathcal {D}=3.1$, $\chi =1$; **b** $\mathcal {D}=3.5$, $\chi =1$; **c** $\mathcal {D}=3.9$, $\chi =1$. For each model, a log power spectral density (PSD) versus log frequency is shown with hedgehog plots for $\mathcal {D}$ and intercepts; 1,048,576 data points

**Fig. 4:**
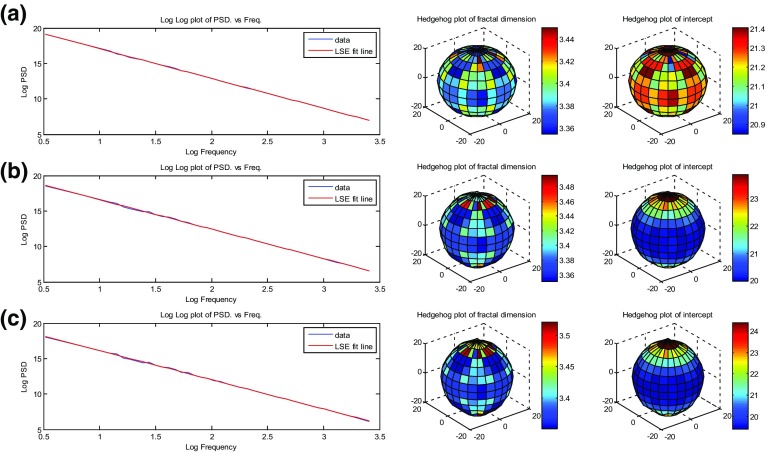
Fractal behaviour tests performed on fractal volumes shown in Fig. 2, where **a** $\mathcal {D}=3.4$, $\chi =1$; **b** $\mathcal {D}=3.4$, $\chi =3$; **c** $\mathcal {D}=3.4$, $\chi =5$. For each model, a log power spectral density (PSD) versus log frequency is shown with hedgehog plots for $\mathcal {D}$ and intercepts; 1,048,576 data points

**Table 1:** Values of tested fractal dimensions of fractal volumes shown in Fig. 1

Volume	Imposed $\chi$	Slope	Imposed $\mathcal {D}$	Tested $\mathcal {D}$	% Error ($\mathcal {D})$ (%)
(a)	1.0	$-$4.806	3.1	3.097	0.097
(b)	1.0	$-$4.005	3.5	3.4975	0.071
(c)	1.0	$-$3.204	3.9	3.8980	0.051

**Table 2:** Values of tested fractal dimensions of fractal volumes shown in Fig. 2

Volume	Imposed $\chi _z$	Slope	Imposed $\mathcal {D}$	Tested $\mathcal {D}$	% Error ($\mathcal {D})$ (%)
(a)	1.0	$-$4.2054	3.4	3.3973	0.079
(b)	3.0	$-$4.153	3.4	3.4235	0.691
(c)	5.0	$-$4.126	3.4	3.4370	1.088

Figures 3 and 4 show some of the main tests carried out on the models shown in Figs. 1 and 2, while Tables 1 and 2 summarise the testing metrics. It can be seen that in all cases, fractal behaviour was obtained very well, with log(magnitude) falling off linearly with log(frequency). In general, the analysed fractal dimensions were very close to that of the imposed fractal dimension (within 0.01 %) when the volumes were isotropic or moderately anisotropic. The effect of the anisotropy is clearly seen in the “hedgehog plot” of intercepts. This hedgehog plot is a plot of intercepts as a function of orientation in three dimensions (i.e. like a rose diagram but in 3-D). Anisotropy in two dimensions would be indicated by an ellipse in a rose diagram and is represented by an ellipsoid in a hedgehog plot. However, the anisotropy would need to be quite large for the subtle differences between ellipsoids to be distinguished. Consequently, the hedgehog plot is also coloured with differences in the colour of each facet of the hedgehog plot referring to the direction perpendicular to that facet, with hotter colours representing greater distances to the central origin of the plot. The shape of the hedgehog plot is a sensitive indicator to the degree and direction of anisotropy. In an isotropic case (Fig. 3), the hedgehog plot of intercepts is nearly a sphere, indicating that the values are almost the same in all direction. However, when one considers the anisotropic cases (Fig. 4), the hedgehog plots of intercepts are now ellipsoids with orientation parallel to the direction of anisotropy. For instance, values of intercepts in Fig. 4 show that they are much higher in the *z* direction than in the *x* and *y* directions. The ratios of the major and minor axis of the hedgehog plot ellipsoids of intercepts infer the degree of anisotropy. It should be noted that as anisotropy increases, there was is an interaction that increases the fractal dimension of the final model, but the effect is very small and falls within an acceptable error (<1 %). The anisotropy needs to be very large in order to have a significant effect on the fractal dimension, and these values of anisotropy are extremely unlikely in real hydrocarbon reservoirs. In all cases, the statistical distributions conformed extremely well to those which were expected in regard to their normality, mean, and standard deviation.

## Reservoir Modelling Procedure

Reservoir property distribution can be modelled by random fractals having self-similar statistics (Hewett 1986). Based on statistical analysis, horizontal variation of sediment accumulation resulting from depositional or erosional processes exhibits statistics similar to fractional Brownian motion (*fBm*). As a result, the horizontal distribution of sediment properties can also be characterised by *fBm* statistics (Zeybek and Onur 2003; Lu et al. 2002; Molz et al. 1997; Liu and Molz 1996; Hewett 1986). Consequently, reservoir properties at reservoir scale that are approximated by and obtained from Gaussian-based fractals using the fBm approach are considered.

### Modelling Reservoir Porosity

The reservoir model is thought of as being composed of a number of layers, *N*. Each layer is composed of voxels, and each voxel has a given porosity that conforms to a given overall porosity distribution that is, for the purposes of this work, assumed to be normal and described by a mean and a standard deviation, as well as a fractal dimension and three anisotropy factors. Since reservoirs are inherently layered due to the way they were initially deposited, the question arises as to what behaviour the porosity statistics of reservoirs shows. Is the porosity fractally distributed in all directions, or only fractally distributed spatially but not vertically? A clear and direct answer to this question could not be found in the literature. However, analysis of well logs shows that while fractal behaviour can be present within particular formations of the same petrofacies in any direction including vertically, a well log of a whole sequence has characteristics similar to fractional Gaussian noise (*fGn*) vertically. This vertical distribution of the characteristics is due to the different behaviours in the different layers that compose the sequence (Sahimi and Yortsos 1990; Hewett 1986). However, since the reservoir is a small unit compared to a sequence of formations, the property distribution is assumed to be fractal both horizontally and vertically, i.e. three-dimensional. As a result, a fractal porosity model can be created by synthesising a volume with a fractal spatial correlation in three dimensions and then renormalising the magnitude of each voxel. The renormalisations scale the magnitude of the fractal volume at each voxel *X*(*x*, *y*, *z*), by the overall porosity distribution to obtain the porosities of each voxel $\phi ( {x,y,z} )$ (Hewett 1986). The scaling could be carried out by ensuring the lowest value of the normalised volume (i.e. zero) is equal to the lowest desired porosity and the highest value of the volume (i.e. unity) is equal to the highest porosity, as follows

13 $$\begin{aligned} \phi \left( {x,y,z} \right) =\phi _\mathrm{min} +\frac{\left[ {X\left( {x,y,z} \right) -X_\mathrm{min} } \right] \left[ {\phi _\mathrm{max} -\phi _\mathrm{min} } \right] }{\left[ {X_\mathrm{max} -X_\mathrm{min} } \right] }, \end{aligned}$$

where $\phi _\mathrm{min}$ and $\phi _\mathrm{max}$ are the desired minimum and maximum porosity values, $X_\mathrm{max}$ and $X_\mathrm{min}$ are the maximum and minimum values of the original fractal volume, *X*(*x*, *y*, *z*) is the value to be renormalised at point *x*, *y*, *z*, and $\phi ({x,y,z})$ is the new renormalised value.

Another way, which was preferred in this study, is to renormalise the mean and standard deviation of the fractal volume to the desired porosity mean and standard deviation. This can be done by first obtaining the standard score (*Z*-score) for each position, which normalises the values to have zero mean a unit variance by Aksoy and Haralick (2001)

14 $$\begin{aligned} Z\left( {x,y,z} \right) =\frac{X\left( {x,y,z} \right) -\mu _X}{\sigma _X }, \end{aligned}$$

where *Z*( *x*, *y*, *z* ) is the Z-score value, $\mu _{X}$ and $\sigma _{X}$ are the overall mean and standard deviation of the fractal volume *X*, which is being normalised. The renormalised porosity volume $\phi ( {x,y,z} )$ can then be obtained by scaling the *Z*-score at each location by the desired mean porosity $\mu _{\phi }$ and its standard deviation $\sigma _{\phi }$

15 $$\begin{aligned} \phi \left( {x,y,z} \right) =\mu _{\phi } +\sigma _{\phi } \times Z\left( {x,y,z} \right) . \end{aligned}$$

### Modelling Reservoir Permeability

Permeability is a primary reservoir property of any reservoir model to be simulated. Each voxel within the reservoir model should be assigned three permeability values, one for each direction (i.e. *x*, *y*, and *z*).

A distinction should be made between the anisotropy of the distribution of fractal permeabilities and the anisotropy in the magnitude of the permeabilities. The anisotropy of the distribution of fractal permeabilities operates at voxel scale and above and controls how “layered” the reservoir permeability model looks. In this study the permeability is calculated from three fractal volumes representing porosity, effective grain size, and cementation exponent using the RGPZ equation (Glover et al. 2006; Glover 2015). It is the anisotropy in each of these volumes $(\chi _{{\phi _{x}}},\chi _{{\phi _{y}}} ,\chi _{{\phi _{z}}} ,\chi _{{d_{x}}}, \chi _{{d_{y}}}, \chi _{{d_{z}}}, \chi _{{m_{x}}}, \chi _{{m_{y}}}$, and $\chi _{{m_{z}}}$) that is ultimately responsible for the development of anisotropy in the calculated permeability volume. In this work, all fractal volumes have lateral anisotropy values which are equal to unity, and a vertical anisotropy greater than unity $(\chi _{{\phi _{x}}} =\chi _{{\phi _{y}}} =\chi _{{d_{x}}} =\chi _{{d_{y}}} =\chi _{{m_{x}}} =\chi _{{m_{y}}} =1;\chi _{{\phi _{z}}} =\chi _{{d_{z}}} =\chi _{{m_{z}}} >1)$, which leads to a significant horizontal layering in the calculated permeability volume.

There is also a microstructural anisotropy which is associated with the fundamental pore structure and arises from the asymmetric operation of depositional and diagenetic processes. The result is a mean vertical permeability that can be up to a factor of 10 smaller than the horizontal permeability. This smaller-scale anisotropy can also be implemented in the model by scaling the vertical permeability to be equal to some defined fraction $\xi$ of the horizontal permeability, where $\xi {=}\overline{k_{z}} /\overline{k_{x}} {=}\overline{k_{z}} /\overline{k_{y}}$, i.e. the mean vertical permeability divided by either of the mean lateral permeabilities, which are equal. For the purposes of this work the horizontal permeability is that calculated from the fractal volumes using the RGPZ method, and $\xi \,{=}\,0.1$.

Consequently, the permeability anisotropy at voxel scale is defined only by the microstructural anisotropy, while the permeability anisotropy at larger scales has contributions both from the microstructural anisotropy and from the larger-scale anisotropy imposed by the fractal volumes.

The RGPZ equation was used to calculate the permeability volume (Glover et al. 2006). This equation is one of the very few “non-empirical” formulae that describe the permeability of a porous medium. The RGPZ equation is given by

16 $$\begin{aligned} {k}=\frac{{d}^{{2}}\phi ^{{3m}}}{{4am}^{\hbox {2}}}, \end{aligned}$$

where *k* is the permeability (in $\hbox {m}^{2})$, *d* is the grain size (in m), $\phi$ is the porosity, *m* is the cementation exponent, and *a* is a constant which usually takes the value $a=8/3$ (Walker and Glover 2010). The RGPZ model requires knowledge of grain size and the cementation exponent as well as the porosity for each voxel in order to calculate the permeability, which is discussed in the next section.

### Modelling Grain Size and Cementation Exponent

We have seen earlier that the distributions of the effective grain size and cementation exponent both behave fractally. Hence, both the grain size and the cementation exponent volumes can be generated in exactly the same manner as the porosity volume, but renormalising with the desired mean and standard deviation for each property. It is possible to create completely new fractal reservoir volumes, one for effective grain size and one for cementation exponent, that are independent of each other and of the porosity volume. Each of these new volumes may have different fractal dimension and anisotropy factors as well as the underlying structures which are defined by the random number seeds used in their construction. However, we have recognised that the relative variation in one physical property, e.g. porosity, is sensitive to the same environmental factors during deposition and diagenesis that also control the grain size and cementation exponent.

The difficulty with generating two volumes for each petrophysical property that share some overall features, but are different, stems from the fact that each random number generator seed used in the fractal generator algorithm produces a completely different and independent fractal volume in an unpredictable fashion (i.e. two consecutive seeds do not produce slightly different volumes). However, in order to overcome this problem and generate volumes that can share some similarities, the following procedure has been used.

Let us consider two random number generators $\mathcal{R}_1$ and $\mathcal{R}_2$ that can be used to generate two independent fractals. For the case that some similarity is required, the two random number generators are mixed linearly as a function of a certain mismatch factor to form a third random number, $\mathcal{R}_3$, according to

17 $$\begin{aligned} \mathcal{R}_3 =\gamma \mathcal{R}_1 +( {1-\gamma } )\mathcal{R}_2, \end{aligned}$$

where ${\gamma }$ is a mismatch factor applied to each Fourier component. So, if $\gamma =1$, 17 collapses to $\mathcal{R}_{3} =\mathcal{R}_1$ and the two volumes will be completely independent to each other, while if $\gamma =0$, 17 collapses to $\mathcal{R}_3 =\mathcal{R}_2$ and the two volumes will match each other exactly. However, if $0<\gamma <1$, $\mathcal{R}_3$ will be a mixture of the two random numbers where smaller value of ${\gamma }$ will produce more similar volumes.

This way, we can control how sensitive a certain property is to another. For example, if we think that the grain size distribution is highly sensitive to porosity distribution, ${\gamma }$ can take values 0.2 or 0.3. However, if we think that cementation exponent is less sensitive to porosity distribution, we can use a value of ${\gamma }=0.5$ or higher.

After generating models for reservoir grain size distribution and cementation exponent, these models are then used in the RGPZ permeability equation (Eq. 16) to calculate the permeability volume for each voxel.

**Fig. 5:**
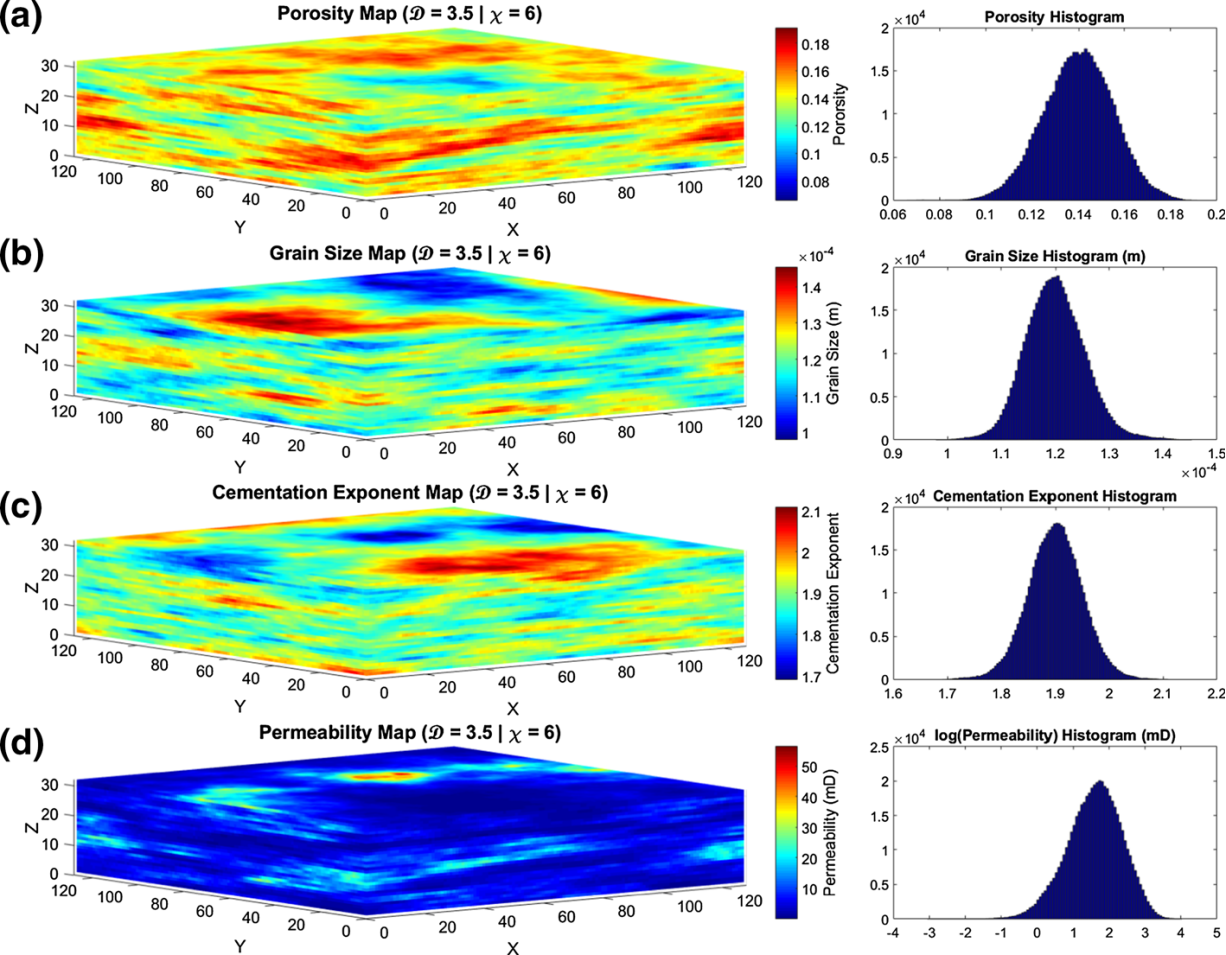
Physical property maps of an example reservoir model with a fractal dimension of $\mathcal {D} = 3.5$, and vertical anisotropy of $\chi =6.0$. **a** porosity, **b** grain size, **c** cementation exponent, and **d** lateral permeability. The *x*, *y*, and *z* axes represent the number of blocks at each direction which are ($128 \times 128 \times 32$; 524,288 voxels) in these particular examples

Figure 5 shows the porosity, grain size, cementation exponent, and lateral permeability maps as a typical example of a reservoir model. The parameters that were used to create the model are given in Table 3.

**Table 3:** Parameters used in the creation of th models shown in Figs. 5 and 6

Physical property	Parameter	Symbol	Value	Unit
Porosity	Fractal dimension	$\mathcal {D}_{{\varphi }}$	3.5	Unitless
	Anisotropy factor	$\chi _{{\varphi }}$	6.0	Unitless
	Distribution arithmetic mean	${\overline{\phi }}$	0.14	Unitless
	Distribution standard deviation	$\Delta \phi$	0.015	Unitless
Grain size (diameter)	Fractal dimension	$\mathcal {D}_\mathrm{d}$	3.5	Unitless
	Anisotropy factor	$\chi _d$	6.0	Unitless
	Distribution arithmetic mean	$\overline{d}$	$120\times 10^{-6}$	m
	Distribution standard deviation	$\Delta d$	$5.5\times 10^{-6}$	m
Cementation exponent	Fractal dimension	$\mathcal {D}_\mathrm{m}$	3.5	Unitless
	Anisotropy factor	$\chi _\mathrm{m}$	6.0	Unitless
	Distribution arithmetic mean	$\overline{m}$	1.9	Unitless
	Distribution standard deviation	$\Delta m$	0.05	Unitless

### Synthetic Poroperm Cross-Plots

**Fig. 6:**
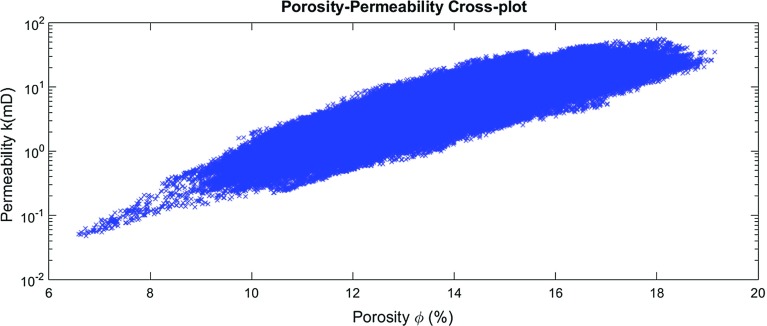
Poroperm cross-plot of the model reservoir shown in Fig. 5 and using parameters given in Table 3; 524,288 data points

Synthetic poroperm^2^ cross-plots were constructed from the reservoir model that is shown in Fig. 5. It is possible to do this for the entire model or for each individual layer. Figure 6 shows the poroperm cross-plot for the entire reservoir. During the modelling it has become clear that the characteristics of the poroperm cross-plot depend upon the standard deviation of reservoir property distribution more than anything else. It is also controlled by the degree of interaction between the three parameters defining the permeability ($\phi$, and *d*). If the cementation factor and the grain size have similar geometrical distribution to porosity, the poroperm cloud will collapse to a single trend curve. More work is needed to be carried out in order to understand these effects. However, it is clear that this approach to reservoir modelling is, in fact, also an approach to poroperm modelling.

### Fluid Saturations and Relative Permeability Modelling

The last major unknown parameter is the fluid saturation of each voxel. The capillary entry pressure of each voxel has been calculated and compared with the capillary pressure it would have for that voxel to contain water or hydrocarbons according to its height above the free water level. For the purposes of this study the free water level was taken as the base of the bottom layer. Accordingly, Brooks and Corey (1966) equation, which describes the capillary pressure as a function of water saturation $P_\mathrm{c} ({S_\mathrm{w}})$, was used. Brooks and Corey (1966) found experimentally that the fluid saturation can be related to capillary and capillary entry pressures by

18 $$\begin{aligned} P_\mathrm{c} =P_\mathrm{e} ( {S_\mathrm{w}^{*}} )^{-\frac{1}{\lambda }}, \end{aligned}$$

where $P_\mathrm{c}$ is the capillary pressure, $P_\mathrm{e}$ is the capillary entry pressure, $S_\mathrm{w}^{*}$ is the reduced saturation of the wetting phase, and $\lambda$ is the pore size distribution index. Although this relation is purely empirical, Li (2004) derived, theoretically, this model from fractal theory of porous media where its fractal dimension $\mathcal {D}$ is related to $\lambda$ by

19 $$\begin{aligned} \lambda =3-\mathcal {D}. \end{aligned}$$

where $2< \mathcal {D} < 3$. Equation 18 can be rearranged to obtain the reduced wetting phase saturation

20 $$\begin{aligned} S_\mathrm{w}^{*} =\left( {\frac{P_\mathrm{e} }{P_\mathrm{c} }} \right) ^{\lambda }. \end{aligned}$$

$S_\mathrm{w}^{*}$ is defined during imbibition of the wetting phase by

21 $$\begin{aligned} S_\mathrm{w}^{*} =\frac{S_\mathrm{w} -S_\mathrm{wr} }{1-S_\mathrm{wr} -S_\mathrm{nwr}}, \end{aligned}$$

and during drainage as

22 $$\begin{aligned} S_\mathrm{w}^{*} =\frac{S_\mathrm{w} -S_\mathrm{wr} }{1-S_\mathrm{wr}}, \end{aligned}$$

where $S_\mathrm{w}$ is the wetting phase saturation, $S_\mathrm{wr}$ is the wetting phase residual saturation, and $S_\mathrm{nwr}$ is the non-wetting phase residual saturation (Li and Horne 2006). In this study, imbibition displacements where a non-wetting invading fluid (oil) is displaced by a wetting fluid (water) are considered.

This approach requires us to know $P_{\mathrm{c}}$ and $P_{\mathrm{e}}$ for each voxel. The capillary pressure in each layer above the free water level is related to the height above the free water layer and the difference in density between the fluids occupying the pores according to

23 $$\begin{aligned} {P}_{\mathrm{c}} {=g}\, \Delta {\rho }\, h, \end{aligned}$$

where *h* is the height above the free water level (in m), *g* is the acceleration due to gravity (taken as $9.81\,\hbox {m}/\hbox {s}^{2}$ in this work), and $\Delta \rho$ is difference in density between the oil and the water (taken as $140\,\hbox {kg}/\hbox {m}^{3}$ in this work).

Independently, the capillary pressure when two immiscible fluids occupy a capillary tube is defined as

24 $$\begin{aligned} {P}_{\mathrm{c}} {=}\frac{{2\sigma \cos \,\theta }}{{r}_{{tube}}}, \end{aligned}$$

where ${\sigma }$ is the interfacial surface tension (taken as 0.035 N/m) and ${\theta }$ is the fluids contact angle (taken as 0 in this work), and $r_\mathrm{tube}$ is the radius of the capillary tube. Assuming that pore throats can be approximated by tubes, and setting $r_\mathrm{tube}=d_\mathrm{pt}/2$, where $d_\mathrm{pt}$ is the effective diameter of the pore throats, Eq. 24 can be rearranged to give the capillary pressure of pore throats as

25 $$\begin{aligned} {P}_{\mathrm{c}} {=}\frac{{4\sigma \cos \,\theta }}{{d}_{{pt}}}, \end{aligned}$$

where $d_\mathrm{pt}$ is the diameter of the pore throats (in m).

The grain diameter can be converted into a pore diameter using the theta transformation (Glover and Walker 2009)

26 $$\begin{aligned} {d}_{{pore}} {=2d}_{{grain}} \sqrt{\frac{{2}\phi ^{{2m}}}{{am}^{{2}}}}, \end{aligned}$$

where $d_\mathrm{pore}$ is the diameter of the pores (in m), $d_\mathrm{grain}$ is the diameter of the grains (in m), and all other symbols have been previously defined. Glover and Déry (2010) extended this conversion to pore throat diameters by defining and calculating a constant conversion for randomly distributed grains, of clastic rocks, such that

27 $$\begin{aligned} {d}_{{pore}} =A\,d_{{pt}}, \end{aligned}$$

where *A* is constant ($A = 1.655$). Equations 15 and 27 can be combined with the definition of capillary pressure for capillary pore throats (Eq. 25) to give

28 $$\begin{aligned} {P}_{\mathrm{c}} =\frac{{2A\sigma \cos \,\theta }}{{d}_{{grain}} }\sqrt{\frac{{am}^{{2}}}{{2}\phi ^{{2m}}}}. \end{aligned}$$

**Fig. 7:**
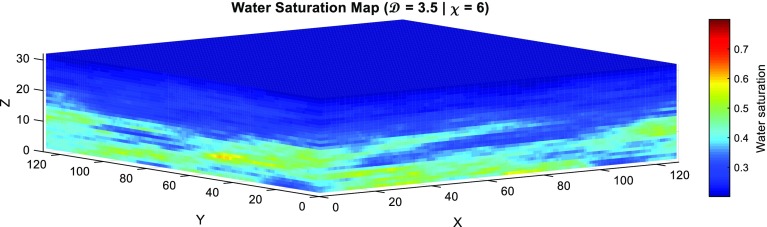
Water saturation map for the model reservoir. The *x*, *y*, and *z* axes represent the number of blocks at each direction which are ($128 \times 128 \times 32$; 524,288 voxels) in this particular example

Similarly, the corresponding capillary entry pressure will be

29 $$\begin{aligned} {P}_{\mathrm{e}} =\frac{{2A\sigma \cos \,\theta }}{{d}_{{largest\,grain}} }\sqrt{\frac{{am}^{{2}}}{{2}\phi ^{{2m}}}}, \end{aligned}$$

where $d_\mathrm{largest\,grain}$ is the largest grain size at each voxel (assumed to be 1.2 bigger than the mean grain size). Equations 23 and 29 can now be used to calculate the capillary pressure ${P}_{\mathrm{c}}$ as a function of height above the free water level and the capillary entry pressure ${P}_{\mathrm{e}}$. The reduced water saturation can now be calculated for each voxel from Eq. 20 if the value of $\lambda$ is known. The water saturation for each voxel can then be calculated using Eq. 21 or 22. In this work, it was assumed that water is the wetting phase fluid, while oil is the non-wetting phase fluid. Hence, water saturation can be calculated by rearranging Eq. 22 as

30 $$\begin{aligned} S_\mathrm{w} =S_\mathrm{w}^{*} \left( {1-S_\mathrm{wr} -S_\mathrm{or} } \right) +S_\mathrm{wr}, \end{aligned}$$

where $S_\mathrm{wr}$ and $S_\mathrm{or}$ are the irreducible water and residual oil saturations, respectively. Figure 7 shows a water saturation map calculated for the reservoir model shown in Fig. 5 for the imbibition case.

**Fig. 8:**
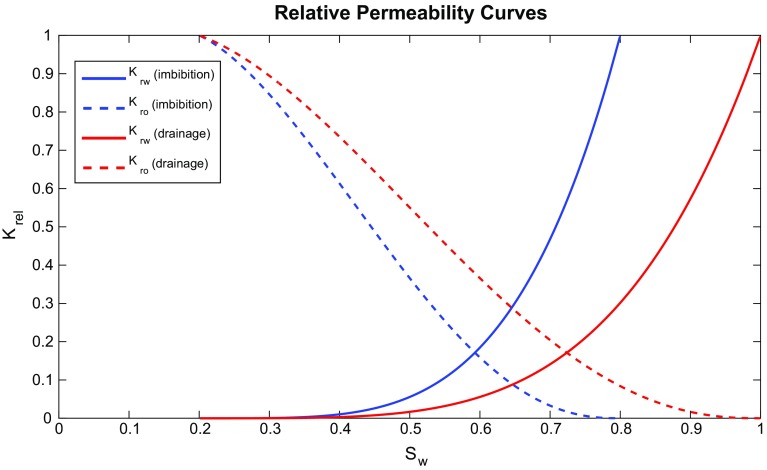
Calculated relative permeability curves using the imbibition and drainage forms of reduced water saturation equations (Eqs. 21, 22)

### Relative Permeability

Synthetic wetting and non-wetting relative permeability curves for each cell of the model were calculated from the reduced water saturation that was calculated in the previous step together with the $\lambda$ value by using the Brooks–Corey–Mualem model (Chen et al. 1999)

31 $$\begin{aligned}&\displaystyle k_\mathrm{rw} =\left( {S_\mathrm{w}^*} \right) ^{\frac{4+5\lambda }{2\lambda }}, \end{aligned}$$

32 $$\begin{aligned}&\displaystyle k_\mathrm{rnw} =\left( {1-S_\mathrm{w}^{*}} \right) ^{\frac{1}{2}}\left[ {1-\left( {S_\mathrm{w}^{*} } \right) ^{\frac{1+\lambda }{\lambda }}} \right] ^{2}. \end{aligned}$$

Figure 8 shows an example of relative permeability curves generated for the reservoir shown in Fig. 5 for both drainage and imbibition cases. In this figure we have imposed irreducible water and residual oil saturations both of 20 %.

### Comparison with Existing Reservoir Modelling Practice

The current practice of reservoir heterogeneity modelling, for the purpose of flow simulation, is generally done by reservoir geostatistical modelling (Pyrcz and Deutsch 2014; Ringrose and Bentley 2015). For example, porosity models can be generated by calculating semivariograms to capture the heterogeneity, and then they can be used in a facies-based sequential Gaussian simulation “sGs” geostatistical algorithm (Al Qassab et al. 2000). It is possible to calculate the variogram of the fractal model and apply sequential Gaussian simulation before comparing the results to a variogram-based model flow. However, given the scope of the paper, this will not be carried out herein.

Standard criteria of reservoir modelling have been established (Pyrcz and Deutsch 2014), including: (i) conditioning the model to data from wells and seismic of the reservoir to be modelled, (ii) heterogeneity, (iii) realisations for uncertainty, (iv) efficiency, and (v) accessibility to reservoir geologists and engineers. The motivations for developing this modelling are to look at the effects of heterogeneity and anisotropy not in a given reservoir but in general terms (i.e. using synthetic data), and so at this stage no effort has been put into conditioning the models to a specific reservoir. The strength of this approach is that the effects of heterogeneity and anisotropy can be examined without the specific characteristics of a given reservoir getting in the way. Although this approach has been developed for looking at heterogeneity and anisotropy in a generic way, research is underway to see whether this type of modelling can be conditioned for use with specific reservoirs and can also be applied to the modelling of fractures within reservoirs, whose length and aperture also follow fractal laws. Additionally, since those models are not stochastic, as explained earlier, there is no uncertainty in such a fully determined model. Moreover, run times depend on how big the analysed model is and on the number of wells used. Regarding the accessibility, it depends on how the final model is integrated into existing software. There are no outstanding issues that need to be overcome before such an approach can begin to be incorporated into existing large-scale software.

In particular, this paper describes the feasibility, mathematical background, and development of the technique together with a few examples. Further examples, including simulation that examines the heterogeneity and anisotropy affects, are the subject of a further paper that is currently being written.

## Modelling Implementation for Test Reservoirs

Reservoir simulations were used in order to quantify the effect of heterogeneity and anisotropy of reservoir properties on oil production. Using the methodology explained earlier, a model with different scenarios of heterogeneity defined by the fractal dimensions ($\mathcal {D}=3.1$, 3.3, 3.5, 3.7, and 3.9) and vertical anisotropy factors ($\chi =1$, 3, and 5) was tested. The model represents a coarse grain, high porosity, and high permeability reservoir having unimodal property distributions.

**Fig. 9:**
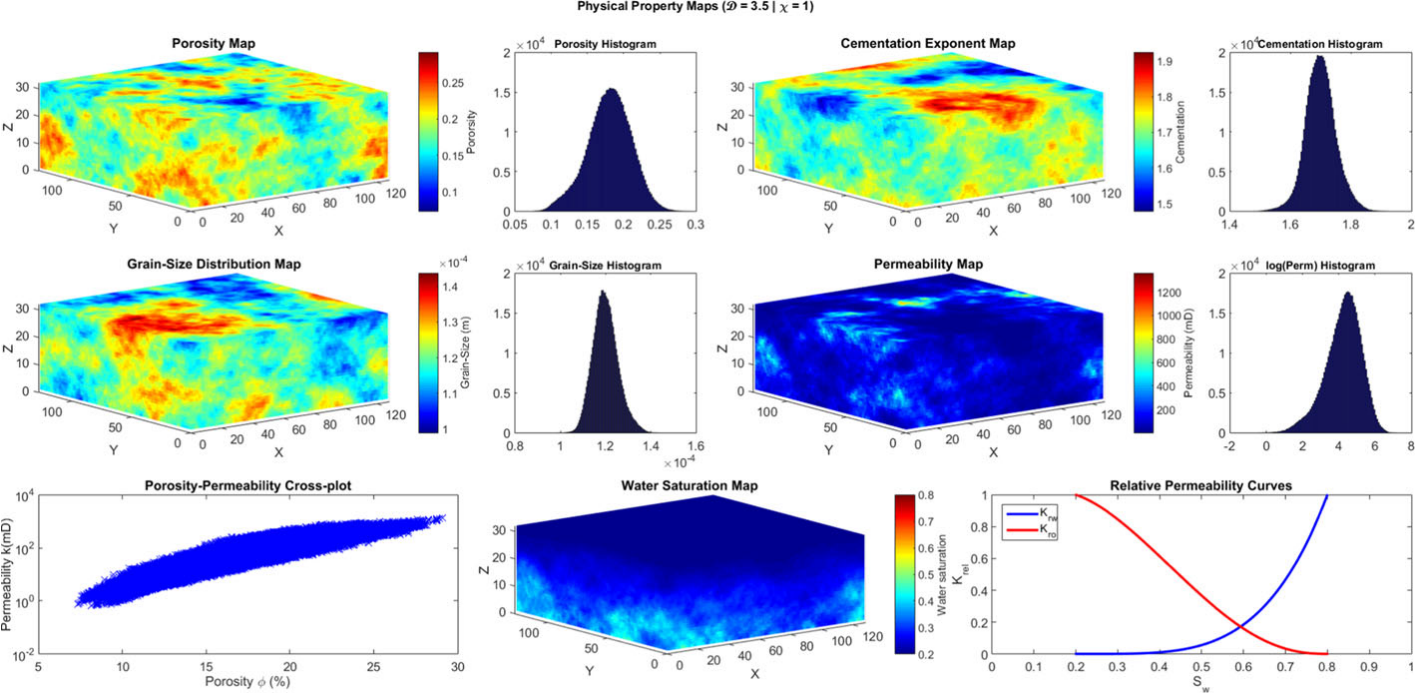
A reservoir model under test representing a coarse-grained reservoir with high porosity and permeability. The parameters that were used to create the model are given in Table 4. This particular case has a fractal dimension $\mathcal {D} = 3.5$ and an anisotropy factor of $\chi = 1.0$. The *x*, *y*, and *z* axes represent the number of blocks at each direction which are ($128 \times 128 \times 32$; 524,288 voxels)

All normalised reservoir volumes that were generated were tested to ensure that their distributions conformed to a Gaussian distribution. Once renormalised each calculated reservoir property (porosity, grain size, and cementation exponent) was also tested for the accuracy of its distributions, that its fractal dimension and anisotropy conformed to that imposed on it, and that the mean value of the reservoir property and its standard deviation matched the desired values. Those volumes which were calculated from the initially modelled reservoir properties such as the permeability and the fluid saturations were also tested to ensure that their distributions, means, standard deviations, fractal dimensions, and anisotropies conformed to what was expected and represented a reasonable description of the physical property in real reservoirs. One of the requirements is that the permeability distribution follows an approximately log-normal distribution, as observed for real reservoirs.

Figure 9 shows one scenario of the tested model, while Table 4 summarises the physical property parameters used for model creation together with the main tests carried out on these parameters. Irreducible water saturation and residual oil saturation of 20 % have been implemented in all cases. Note that the pore size distribution index has been fixed to 1.2 at all scenarios.

**Table 4:** Parameter summary of the reservoir shown in Fig. 9

Physical property	Parameter	Symbol	Imposed value	Tested value	% Error
Porosity	Fractal dimension	$\mathcal {D}_{{\varphi }}$	3.5	3.497	0.08
	Anisotropy factor	$\chi _{{\varphi }}$	1.0	NA	NA
	Distribution peak	$\overline{\phi }$	0.18	0.18	0
	Distribution standard deviation peak	$\Delta \phi$	0.03	0.03	0
Grain size (diameter)	Fractal dimension	$\mathcal {D}_\mathrm{d}$	3.5	3.535	1.0
	Anisotropy factor	$\chi _d$	1.0	NA	NA
	Distribution peak	$\overline{d}$	$120\,\upmu \hbox {m}$	$120\,\upmu \hbox {m}$	0
	Distribution standard deviation peak	$\Delta d$	$5.5\,\upmu \hbox {m}$	$5.5\,\upmu \hbox {m}$	0
Cementation exponent	Fractal dimension	$\mathcal {D}_\mathrm{m}$	3.5	3.456	1.2
	Anisotropy factor	$\chi _\mathrm{m}$	1.0	NA	NA
	Distribution peak	$\overline{m}$	1.7	1.7	0
	Distribution standard deviation peak	$\Delta m$	0.05	0.05	0
Lateral permeability	Fractal dimension	$\mathcal {D}_{k}$	Not defined	3.459	
	Anisotropy factor	$\chi _k$		NA	
	Distribution peak	$\overline{k}$		103	
	Distribution standard deviation peak	$\Delta \phi$		98.4	

Also, testing data for the reservoir model implemented with a fractal dimension $\mathcal {D} = 3.5$ and an anisotropy factor of $c= 1.0$ are shown

*NA* no analysis tool is available to test it

## Initial Simulation Results

Simulation of the models shown in Fig. 9 was carried out using the finite difference $\hbox {Tempest}^{{\textregistered }}$ simulator (version 7.0.4) on a distributed $\hbox {Windows}^{\mathrm{TM}}$ 7 platform at the University of Leeds. The initial fluid pressure and temperature were taken to be 2300 psi and $120\,^{\circ }\hbox {C}$ to be in accordance with those typical values for real reservoirs. The model was composed of 32 layers each 3 m thick. Each layer was composed of $128\times 128$ cells which were 100 m by 100 m in lateral extent, making up a total of 524,288 voxels, extending 12.8 km in each lateral direction and 96 m vertically.

**Fig. 10:**
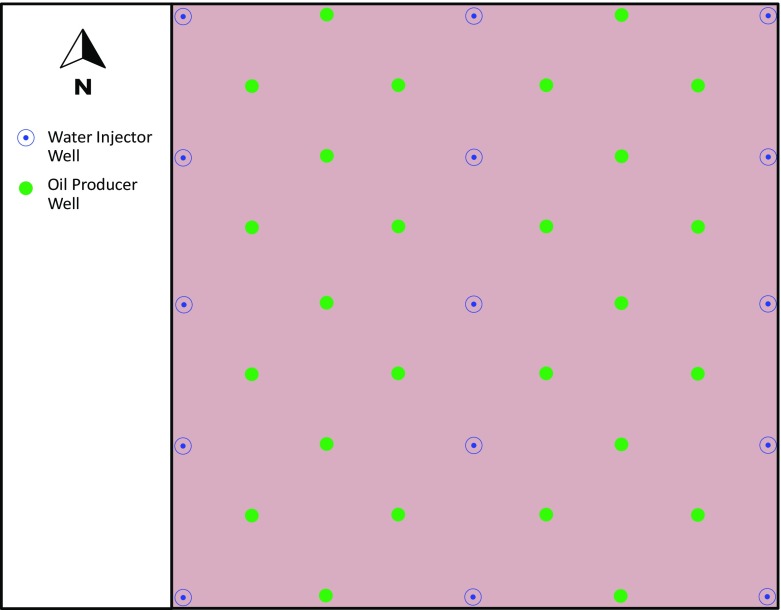
Well pattern map

The solution was run with a black oil model simulator over 60 years. Dynamic simulations on our models invoke water flooding with 15 vertical injectors injecting at a constant rate of $5000\,\hbox {m}^{3}/\hbox {day}$ at oil industry standard conditions (1 atmosphere pressure and $60\,^{\circ }\hbox {F}$ or $15.55\,^{\circ }\hbox {C}$), and a total of 26 vertical producers producing at a constant rate of 2000 standard $\hbox {m}^{3}/\hbox {day}$ distributed as shown in Fig. 10. Water injection was used to support the reservoir fluid pressure from the start until the end of the simulation. Producers and injectors have constant bottom hole pressure of 10,000 psi, respectively.

The following parameters were simulated: (i) reservoir fluid pressure, (ii) oil production, (iii) gas production, (iv) water production, and (v) mean relative permeability differential, both as reservoir maps and as a function of time. Simulations were initially run for five different fractal dimensions ($\mathcal {D}=3.1$, 3.3, 3.5, 3.7, and 3.9) and hence five different reservoir heterogeneities each at a fixed vertical anisotropy factor ($\chi =1$, 3, and 5), leaving all other parameters constant.

**Fig. 11:**
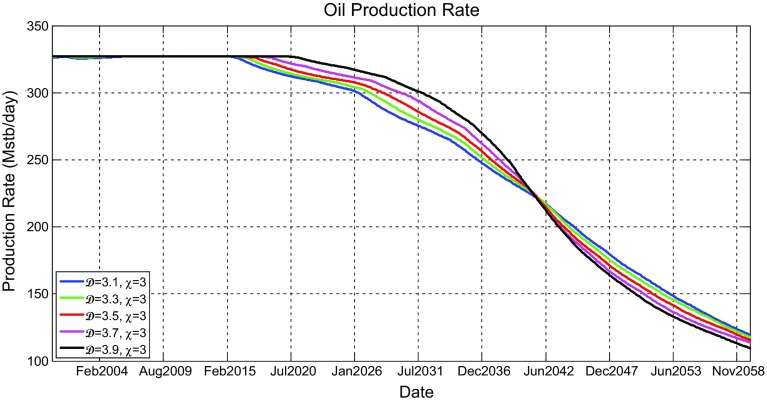
Simulated oil production rates (Mstb/day) as a function of time (years) for the reservoir model having five different fractal dimensions ($\mathcal {D} = 3.1$, 3.3, 3.5, 3.7, and 3.9) and a fixed vertical anisotropy factor of $\chi =3$

Figure 11 shows the oil production rate profiles for the models shown in Fig. 9, which reveal that the production rates gradually decrease after few years of initial production plateau initiated as a result of the imposed oil production targets on production wells in all scenarios. It can be seen that the oil production rates for higher fractal dimensions have initial plateau that last little longer, but once they begin to decline, they decline faster. This was revealed in all scenarios. Although this occurs, it was noticed that increasing the heterogeneity has little effect on oil production.

**Fig. 12:**
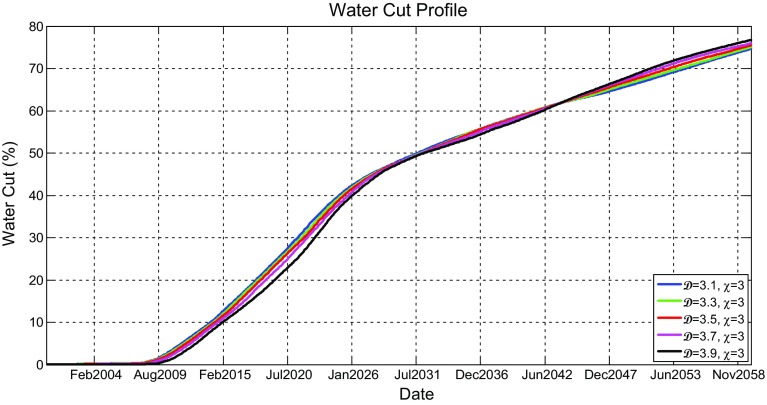
Simulated water cut (%) for all producers as a function of time (years) for reservoir-1G having five different fractal dimensions ($\mathcal {D} = 3.1$, 3.3, 3.5, 3.7, and 3.9) and a fixed vertical anisotropy factor of $\chi =3$

Figure 12 shows the simulated water cut percentage as a function of production time. All water cut graphs show that production of water would start only after about 4 years of production and would increase until the end of the simulation period. They also show that water production would be rapid in the first few months and then become gradual. It can be seen that different scenario profiles are really not affected by $\mathcal {D}$.

**Fig. 13:**
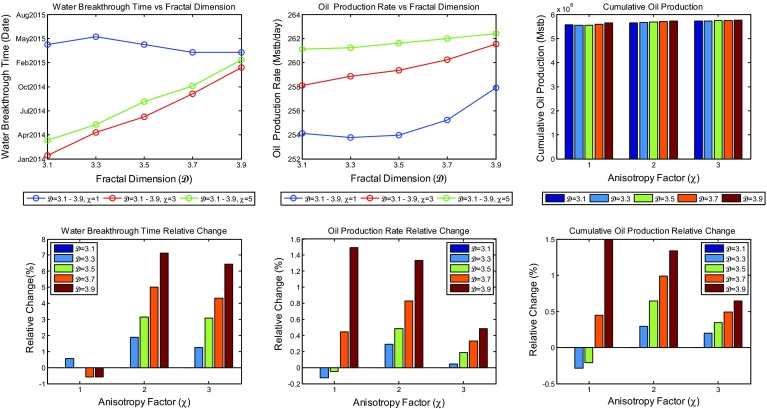
A combined plot showing the change of the water breakthrough times, the average oil production rate, and the cumulative oil production with fractal dimension and anisotropy factor of the reservoir model

The effect of water cut is best analysed by breakthrough times, which was taken by us to be a water cut of 10 %. Combined plots showing the change of water breakthrough times, average oil production rate, and cumulative oil production over the production time as a function of heterogeneity and anisotropy for the reservoir together with their relative change as compared to the most homogeneous scenarios are shown in Fig. 13. Analysis of these plots shows that neither the overall average rate of oil production nor the overall cumulative oil production is greatly affected by the increase in heterogeneity as the change of the simulated values at different heterogeneities is very small, with a general trend of a slight increase in overall average production rate, and hence oil cumulative production, as the heterogeneity increases. The model showed earlier breakthrough times as the increase in heterogeneity was applied. Nevertheless, the shift in water breakthrough times with different degrees of heterogeneity does not exceed few months. The difference in breakthrough times even decrease more as the anisotropy increases, suggesting that there is probably an interaction between the heterogeneity and anisotropy at high values that decrease the effect of each other.

From the above reporting of all tested reservoir realisations, it may be said that the variation of the fractal dimension from 3.1 to 3.9 that determines the heterogeneity of the distribution have an insignificant effect on cumulative oil production and oil production rates.

The insensitivity of these reservoir oil production rates to a change in the fractal dimension is related to the nature of the generated fractal volumes. The fluid displacement through fractal volumes depends on the imposed mean values of porosity and permeability generated by *fBm* algorithm. As seen before, the shape of the models is defined by (i) seeding the random number generator used in the *fBm* generation algorithm, and (ii) the fractal dimension, which can take the values $3< \mathcal {D} < 4$. If the created *fBm* realisation is considered to reflect all the available geologic information (i.e. similar to having enough data to describe a reservoir accurately), changing the fractal dimension of a model does not change the overall shape of the model. In other words, if any two points in the models are considered, the overall geometrical distribution between them does not change, and hence the effective transport property values between them does not change. The only thing that changes is the irregularity of the distribution. This irregularity makes it harder for the oil to pass through, and hence longer time plateau, but not with great difference if compared with smoother models because the effective porosity and permeability values between those two points are almost the same at all heterogeneities. Thus, providing that the reservoir properties were mapped accurately, the value of the fractal dimension will not have a significant effect. The problem with real-world heterogeneous reservoirs is the insufficiency of data available to produce a good model describing the heterogeneous behaviour between the wells.

The previous results are consistent with the findings by Aasum et al. (1991) and Babadagli (1999), in that varying $\mathcal {D}$ only is not significant, but contradicts the conclusions made by Li and Xie (2011) which shows that $\mathcal {D}$ has a great effect. Aasum et al. (1991) synthesised a 2-D fractal cross section where porosity and permeability were fractally interpolated between two wells where they were measured. Then, they studied the effect of $\mathcal {D}$ on the miscible displacement process and found it is not significant. Alternatively, Babadagli (1999) generated synthetic 1-D *fBm* distributions where he normalised to porosity logs before converting them to permeability logs using an exponential correlation formula ($k=a10^{\Phi b})$. After that, he generated horizontal cross sections assuming no change horizontally and studied the effect of $\mathcal {D}$ on production performance of these cross sections. He noted that $\mathcal {D}$ has little effect.

Obviously, these two studies used 2-D and 1-D *fBm*, respectively, and assumed nothing is changing other than $\mathcal {D}$, which they found to be not significantly affecting reservoir’s performance.

In contrast, Li and Xie (2011) did it the other way around. They studied the effect of $\mathcal {D}$ on production performance of reservoirs in Hailar Basin, China. They obtained the fractal dimension of core samples from 7 wells across the reservoir, with measured values ranging between 2.36 and 2.72, using the mercury intrusion experiment. Then, they analysed the production history of the wells and noted a clear correlation between the fractal dimension of samples and the oil production and water cut of wells, such that oil wells happened to be drilled in higher fractal dimension reservoirs had the smallest monthly production rate and shortest breakthrough times. This conclusion agrees very well with the industry experience, as it is well known that heterogeneous reservoirs are less efficient when compared to homogeneous ones. Therefore, there must be something other than the fractal dimension, or occurred as a result of the fractal dimension, that affected the reservoir performance. Note that the fractal dimension defines the heterogeneity at microscopic scale, while the reservoir performance is measured at macroscopic scale.

One possibility might be related to fractal capillary pressure. Note that all the previous models were simulated assuming the same value of the pore size distribution index ($\lambda$) used in Corey–Brooks capillary pressure relation implemented in water saturations calculation (Eq. 18), resulting in water saturation volumes that share almost the same mean values and overall geometrical distribution at all heterogeneities.

Nevertheless, in real reservoirs, this might not be the case. Our intension on the previous tests was to study the effect of accuracy on the porosity and permeability modelling while assuming everything else is constant. Yet, it is well known that the heterogeneity affects not only the variation of the distribution, but more importantly, the capillary forces and fluids saturation across the reservoir. As the heterogeneity increases, the capillary pressure changes across the reservoir, causing higher irreducible water saturations. This reveals another reason for heterogeneity being insignificant in the previous tests.

Equation 19 clarifies this concept, as ($\lambda )$ is related to ($\mathcal {D}$), showing that porous media with greater heterogeneity have smaller values of $\lambda$ (Li 2010; Standing 1975). Thus, a relationship between ($P_\mathrm{c}$) and ($\mathcal {D}$) can be inferred. If the natural logarithm of Eq. 18 is obtained, we get

33 $$\begin{aligned} \log P_\mathrm{c} =-\frac{1}{{\lambda }}\log S_\mathrm{w}^{*}+\log P_\mathrm{e} \end{aligned}$$

**Fig. 14:**
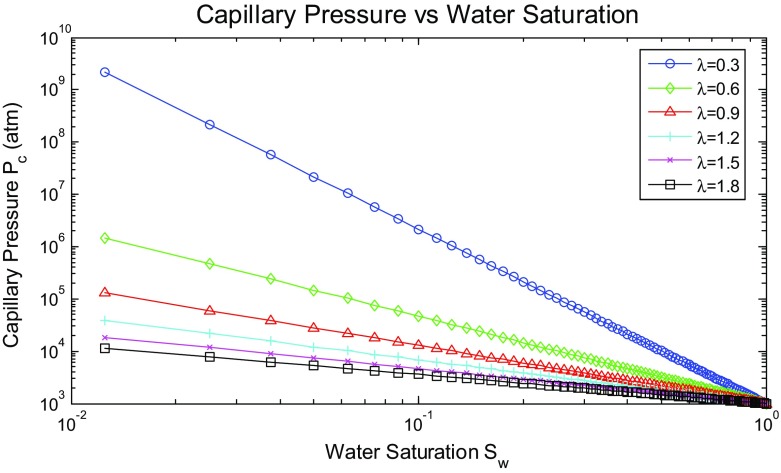
A plot of capillary pressure as a function of water saturation having different values of pore size distribution index $\lambda$. Note that the intercept at 100 % $S_\mathrm{w}$ reveals the capillary entry pressure

Equation 33 foresees that a log–log plot of $P_\mathrm{c}$ as a function of $S_\mathrm{w}^{*}$ reveals a linear relationship with a slope of ($-1/\lambda )$, and the intercept at 100 % $S_\mathrm{w}^{*}$ is $P_\mathrm{e}$. Figure 14 provides type curves generated using Eq. 33, showing that steeper slopes result from higher $\lambda$ values, meaning large variation in pore sizes, and hence poorer-quality reservoirs (Standing 1975). Additionally, higher $\lambda$ values imply an upward shift of the fitting curve, and hence greater variations in the capillary pressure, resulting in higher $S_\mathrm{wr}$ values, associated with heterogeneous reservoirs.

**Fig. 15:**
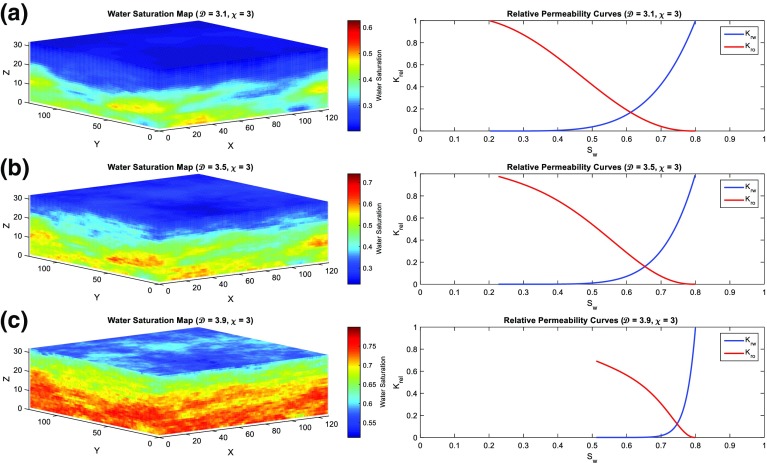
A test of calculating $S_\mathrm{w}$ with varying $\lambda$ according to $\mathcal {D}$ using Eqs. 19, 20, and 30, where **a** $\mathcal {D}=3.1$; **b** $\mathcal {D}=3.5$; and **c** $\mathcal {D}=3.9$. The water saturation (*left*) calculation will differ significantly as the heterogeneity increases with higher $S_\mathrm{wr}$ as shown in the relative permeability curves (*right*)

If the relation between $\lambda$ and $\mathcal {D}$ is taken into account, the water saturation calculation will differ significantly as the heterogeneity increases, where higher $S_\mathrm{wr}$ are obtained. Figure 15 shows water saturation volumes and the relative permeability curves of the reservoir model calculated with $\lambda$ being a function of $\mathcal {D}$. Figure 15 shows clearly that the fractal dimension imposed on the reservoir by the modelling process controls the model’s heterogeneity, and how the heterogeneity, in turn, control the spatial distribution of the initial fluids saturation.

**Fig. 16:**
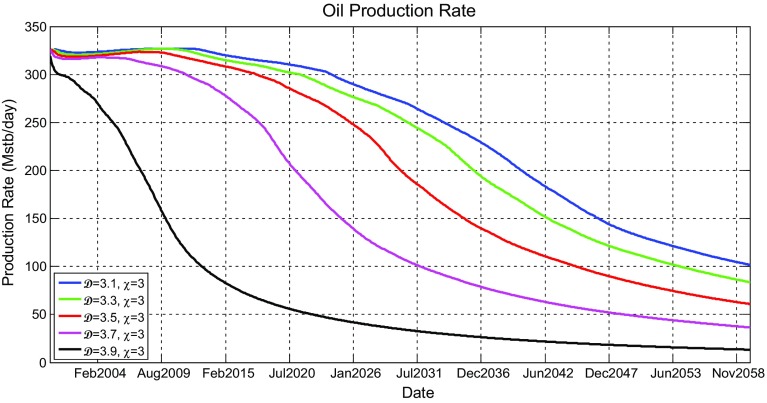
Simulated oil production rates (Mstb/day) as a function of time (years) for the reservoir model having five different fractal dimensions ($\mathcal {D} = 3.1$, 3.3, 3.5, 3.7, and 3.9) and a fixed vertical anisotropy factor of $\chi =3$

**Fig. 17:**
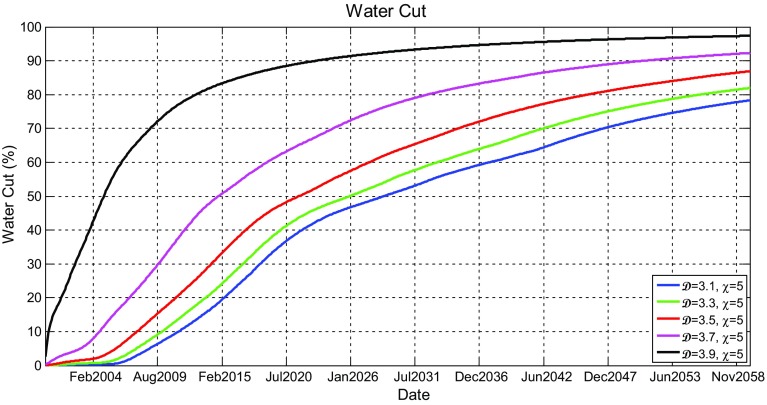
Simulated water cut (%) for all producers as a function of time (years) for the reservoir model having five different fractal dimensions ($\mathcal {D} = 3.1$, 3.3, 3.5, 3.7, and 3.9) and a fixed vertical anisotropy factor of $\chi =3$

**Fig. 18:**
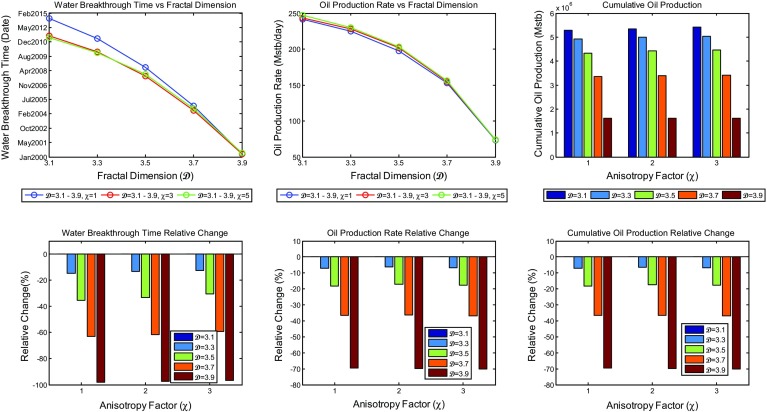
A combined plot showing the change of the water breakthrough times, the average oil production rate, and the cumulative oil production with fractal dimension and anisotropy factor of the reservoir model after the modification

With this new implementation, the effect of heterogeneity on reservoir production performance was reanalysed. New simulation results of oil production rate and water cut profiles are shown in Figs. 16 and 17, respectively. Different scenarios of heterogeneity at fixed anisotropies are shown. Moreover, combined plots of the onset of significant water production, the average oil production rates, and overall cumulative oil production over the production time together with their relative change compared to the homogeneous case are shown in Fig. 18.

It is immediately clear that the reservoir heterogeneity has now a huge impact upon all main measures of reservoir performance. The hydrocarbon production declines sharply 2 years after the start of production for the most heterogeneous reservoir ($\mathcal {D}=3.9$) and is different by over 10 % one year after the start of production and reaches 40 % 8 years later. Additionally, as shown in Fig. 18, the cumulative hydrocarbon production significantly decrease as the heterogeneity increases. Furthermore, breakthrough times change enormously depending on the fractal dimension used (e.g. BT is earlier by 13 years when $\mathcal {D}=3.9$ compared to $\mathcal {D}=3.1$ when $\chi =1$).

Thus, based on these results, a clear correlation between reservoir’s fractal dimension and its performance is revealed such that reservoirs with higher microscopic heterogeneity yield less hydrocarbon production and greater water cut with early water breakthrough times. This does not only result from the irregularities introduced by heterogeneity, but is more importantly because of the consequence effect of the heterogeneity on the capillary pressure. These results now agree very well with the findings presented by Li and Xie (2011). These are very significant uncertainties for a hydrocarbon company if the heterogeneity of their reservoir is not well defined. On this basis, the full effects of heterogeneity and anisotropy have to be addressed before modelling and simulation is carried out.

## Conclusions

This paper has shown how the fractal concept can be used to construct a generic heterogeneous and anisotropic reservoir model that reproduces faithfully the static and spatial characteristics that real heterogeneous and anisotropic reservoirs exhibit. The models are not stochastic, but are completely reproducible when the same random number seeds are used. Different runs using different random number seeds, but with the same physical characteristics allow a population of model reservoirs to be built so that the natural variability and uncertainty in reservoir simulation results may be quantified.

In particular, this paper has evolved a method for generating synthetic (reproducible) three-dimensional reservoir volumes in which the heterogeneity and anisotropic vertically and/or laterally are controllable using imposed fractal dimensions, and the anisotropy is controlled using an anisotropy factor in any direction, respectively. In these models each voxel is correlated to each of the surrounding voxels in a fractal manner. Testing of the fractal volumes shows that the values are spatially and vertically correlated according to the imposed fractal dimension and anisotropy factors to within a very small error.

Basic simulation of the main measures of reservoir performance including the reservoir fluid pressure, hydrocarbon production rates, and the timing of water breakthrough on reservoir models representing heterogeneous reservoirs under test, with a range of heterogeneities (fractal dimension) between 3.1 and 3.9, show that oil production and water encroachment are controlled by permeability heterogeneity, vertical anisotropy, placement of the wells, and, more importantly, the capillary pressure heterogeneity. In fact, vertical displacement depends on the permeability of the most impermeable layer.

Supposing that the generated fractal volumes represent all the available geologic information of a reservoir, and while the change considered is only heterogeneity and anisotropy of the property distributions only (i.e. other parameters are not affected), both the heterogeneity and anisotropy have little effect on reservoirs performance. Thus, providing that the overall property distributions were mapped with good accuracy, the heterogeneity and anisotropy have small effect.

Conversely, if the effect of the heterogeneous spatial distribution of reservoir properties, which is fractal, on other reservoir parameters such as reservoir capillary pressure is taken into account, heterogeneity can significantly, and destructively, impact the reservoir performance behaviour. Under such circumstances, a clear correlation between the fractal dimension and the simulation results is noted such that reservoirs with greater heterogeneity have less oil production rate, greater water cut, earlier water breakthrough time, faster production decline, and faster pressure drop. This is due to the fact the heterogeneity causes the capillary pressure to vary across the reservoir, leading to high connate water saturations and greater water channelling. This shows that the heterogeneity itself, at pore scale, in terms of how the physical properties are distributed in the reservoir is not significant. However, the heterogeneity consequences are significant if not taken into account (e.g. capillary pressure variation).

This approach to modelling a reservoir is not suited to describing the specifics of any given reservoir, but can help us understand the size of the uncertainties in simulation results that arise from either not knowing or not taking into account the full degree of heterogeneity and anisotropy a reservoir exhibits. Given that the majority of modern reservoirs are both significantly heterogeneous and/or anisotropic, it is important to quantify these effects proactively.

## List of symbols

$\mathcal {D}$: The fractal dimension
*n*: The Euclidean dimension
$\chi$: The anisotropy factor
$$\phi $$: Porosity
*k*: Absolute permeability
$$k_\mathrm{r}$$: Relative permeability
$$S_\mathrm{w}$$: Water saturation
$S_\mathrm{wr}$: Residual (irreducible) water saturation
$S_\mathrm{or}$: Residual oil saturation
$S_\mathrm{w}^{*}$: Reduced water saturation
$\uplambda$: Pore size distribution index
$\upbeta$: The spectral exponent
*H*: The Hurst exponent (dimension)
$\upsigma$: Surface or interfacial tension
$\uptheta$: Fluid contact angle
$$\uptau $$: Tortuosity
*m*: Cementation exponent
*d*: Grain size
$$P_\mathrm{c}$$: Capillary pressure
$P_\mathrm{e}$: Capillary entry pressure
*f*: Frequency
*k*, *l*, and *w*: The directional frequencies corresponding to the *x*, *y*, and *z*
$\mathcal{R}$: A random number generator
$\gamma$: A mismatch factor between two independent fractal volumes
Mstb/day: Thousand stock tank barrels per day
Mstb: Thousand stock tank barrels
MMstb: Million stock tank barrels
psia: Pounds per square inch absolute
*g*: Gravitational acceleration
*h*: The height above the free water level
$$\rho $$: Fluids density
Poroperm cross-plots: Porosity–permeability cross-plots

## Footnotes

1 The distribution of resultant volume value magnitudes is defined by the type of distribution from which the random number seed samples random numbers (e.g. the use of normally distributed random numbers produce a surface whose heights are normally distributed too). For the purpose of this study, normal distributions are implemented because they are more likely to simulate distribution of reservoir properties in reality.

2 Poroperm stands for porosity–permeability.

## References

[CR1] Aasum Y, Kelkar MG, Gupta SP (1991). An application of geostatistics and fractal geometry for reservoir characterization. SPE Form. Eval..

[CR2] Aksoy S, Haralick RM (2001). Feature normalization and likelihood-based similarity measures for image retrieval. Pattern Recognit. Lett..

[CR3] Al Qassab, H.M., Fitzmaurice, J., Al-Ali, Z.A., Al-Khalifa, M.A., Aktas, G., Glover, P.W.J.: Cross-discipline integration in reservoir modeling: the impact on fluid flow simulation and reservoir management. Paper Presented at the SPE Annual Technical Conference and Exhibition (2000)

[CR4] Al-Ali, Z.A., Al-Qassab, H.M.: Optimizing simulation models by upscaling from integrated reservoirs models; a case history. SPE Asia Pacific Conference on Integrated Modelling for Asset Management, pp. 25–26 (2000)

[CR5] Alqassab, H.M., Heine, C.J.: A geostatistical approach to attribute interpolation using facies templates, an advanced technique in reservoir characterization. Paper Presented at the Abu Dhabi International Petroleum Exhibition and Conference (1998)

[CR6] Babadagli T (1999). Effect of fractal permeability correlations on waterflooding performance in carbonate reservoirs. J. Pet. Sci. Eng..

[CR7] Brooks, R.H., Corey, A.T.: Properties of porous media affecting fluid flow. Paper Presented at the Journal of the Irrigation and Drainage Division, Proceedings of the American Society of Civil Engineers (1966)

[CR8] Brown, S.R.: Simple mathematical model of a rough fracture. J. Geophys. Res.: Solid Earth **100**, 5941–5952 (1995)

[CR9] Chen J, Hopmans J, Grismer M (1999). Parameter estimation of two-fluid capillary pressure-saturation and permeability functions. Adv. Water Resour..

[CR10] Dimri VP, Srivastava RP, Vedanti N (2012). Fractal Models in Exploration Geophysics: Applications to Hydrocarbon Reservoirs.

[CR11] Family F, Vicsek T (1991). Dynamics of Fractal Surfaces.

[CR12] Glover PWJ (2015). Geophysical properties of the near surface Earth: electrical properties. Treatise Geophys.

[CR13] Glover PWJ, Déry N (2010). Streaming potential coupling coefficient of quartz glass bead packs: dependence on grain diameter, pore size, and pore throat radius. Geophysics.

[CR14] Glover PWJ, Walker E (2009). Grain-size to effective pore-size transformation derived from electrokinetic theory. Geophysics.

[CR15] Glover PWJ, Matsuki K, Hikima R, Hayashi K (1997). Fluid flow in fractally rough synthetic fractures. Geophys. Res. Lett. Geophys. Res. Lett..

[CR16] Glover PWJ, Matsuki K, Hikima R, Hayashi K (1998). Fluid flow in synthetic rough fractures and application to the Hachimantai geothermal hot dry rock test site. J. Geophys. Res. Solid Earth.

[CR17] Glover PWJ, Zadjali II, Frew KA (2006). Permeability prediction from MICP and NMR data using an electrokinetic approach. Geophysics.

[CR18] Hewett, T.A.: Fractal distributions of reservoir heterogeneity and their influence on fluid transport. In: SPE Annual Technical Conference and Exhibition (1986)

[CR19] Hewett, T.A.: Modelling reservoir heterogeneity with fractals. Quant. Geol. Geostat. Geostat. Tróia **’92**, 455–466 (1993)

[CR20] Hewett, T. A.: Modeling reservoir heterogeneity using fractals. In: AGU Fall Meeting Abstracts vol. 1, pp. 3. (2001)

[CR21] Hewett TA, Behrens RA (1990). Conditional simulation of reservoir heterogeneity with fractals. SPE Formation Eval..

[CR22] Isakov E, Ogilvie SR, Taylor CW, Glover PWJ (2001). Fluid flow through rough fractures in rocks I: high resolution aperture determinations. Earth Planet. Sci. Lett..

[CR23] Katz AJ, Thompson AH (1985). Fractal sandstone pores: implications for conductivity and pore formation. Phys. Rev. Lett. Phys. Rev. Lett..

[CR24] Krohn CE (1988). Fractal measurements of sandstones, shales, and carbonates. J. Geophys. Res..

[CR25] Krohn CE (1988). Sandstone fractal and Euclidean pore volume distributions. J. Geophys. Res..

[CR26] Krohn CE, Thompson AH (1986). Fractal sandstone pores: automated measurements using scanning-electron-microscope images. Phys. Rev. B.

[CR27] Li, K.: Characterization of rock heterogeneity using fractal geometry. In: Proceedings of SPE International Thermal Operations and Heavy Oil Symposium and Western Regional Meeting (2004)

[CR28] Li K (2010). Analytical derivation of Brooks-Corey type capillary pressure models using fractal geometry and evaluation of rock heterogeneity. J. Pet. Sci. Eng..

[CR29] Li, K., Horne, R.N.: Comparison of methods to calculate relative permeability from capillary pressure in consolidated water-wet porous media. Water Resour. Res. **42** (2006)

[CR30] Li, K., Xie, R.: Effect of heterogeneity on production performance in low permeability reservoirs. In: SPE EUROPEC/EAGE Annual Conference and Exhibition (2011)

[CR31] Liu H, Molz F (1996). Discrimination of fractional Brownian movement and fractional Gaussian noise structures in permeability and related property distributions with range analyses. Water Resour. Res..

[CR32] Lozada-Zumaeta M, Arizabalo RD, Ronquillo-Jarillo G, Coconi-Morales E, Rivera-Recillas D, Castrejón-Vácio F (2012). Distribution of petrophysical properties for sandy-clayey reservoirs by fractal interpolation. Nonlinear Process. Geophys..

[CR33] Lu S, Molz FJ, Fogg GE, Castle JW (2002). Combining stochastic facies and fractal models for representing natural heterogeneity. Hydrogeol. J..

[CR34] Mandal, D., Tewari, D.C., Rautela, M.S., Misra, T.R.: Use of fractal geometry for determination of pore scale rock heterogeneity. In: International Conference & Exposition on Petroleum Geophysics, Kolkata (2006)

[CR35] Mandelbrot BB (1977). Fractals: Form, Chance, and Dimension.

[CR36] Mandelbrot BB, Van Ness JW (1968). Fractional Brownian motions, fractional noises and applications. SIAM Rev..

[CR37] Molz F, Liu H, Szulga J (1997). Fractional Brownian motion and fractional Gaussian noise in subsurface hydrology: a review presentation of fundamental properties, and extensions. Water Resour. Res..

[CR38] Ogilvie SR, Isakov E, Glover PWJ (2002). Advances in the characterization of rough fractures in hydrocarbon reservoirs. First Break..

[CR39] Ogilvie SR, Isakov E, Taylor CW, Glover PWJ (2003). Characterization of rough-walled fractures in crystalline rocks. Geol. Soc. Lond. Spec. Publ..

[CR40] Ogilvie SR, Isakov E, Glover PWJ (2006). Fluid flow through rough fractures in rocks. II: a new matching model for rough rock fractures. Earth Planet. Sci. Lett..

[CR41] Perez G, Chopra AK (1997). Evaluation of fractal models to describe reservoir heterogeneity and performance. SPE Form. Eval..

[CR42] Pyrcz MJ, Deutsch CV (2014). Geostatistical Reservoir Modelling.

[CR43] Rieu M, Sposito G (1991). Fractal fragmentation, soil porosity, and soil water properties: I. Theory. Soil Sci. Soc. Am. J..

[CR44] Ringrose P, Bentley M (2015). Reservoir Model Design: A Practitioner’s Guide.

[CR45] Russ JC (1994). Fractal Surfaces.

[CR46] Sahimi, M., Yortsos, Y.C.: Applications of fractal geometry to porous media: a review. In: Annual Fall Meeting of the Society of Petroleum Engineers, p. 3, New Orleans, LA (1990)

[CR47] Saupe D, Peitgen H-O, Saupe D, Barnsley MF (1988). Algorithms for random fractals. The Science of Fractal Images.

[CR48] Shen, P., Liu, M. and Jia, F.: Application of Fractal Techniques in Reservoir Development. In: SPE International Oil and Gas Conference and Exhibition in China. Society of Petroleum Engineers (1998)

[CR49] Standing MB (1975). Notes on Relative Permeability Relationships.

[CR50] Thompson AH (1991). Fractals in rock physics. Annu. Rev. Earth Planet. Sci..

[CR51] Thompson AH, Katz AJ, Krohn CE (1987). The microgeometry and transport properties of sedimentary rock. Adv. Phys..

[CR52] Turcotte DL (1997). Fractals and Chaos in Geology and Geophysics.

[CR53] Tyler SW, Wheatcraft SW (1990). Fractal processes in soil water retention. Water Resour. Res..

[CR54] Voss RF, Peitgen H-O, Saupe D, Barnsley MF (1988). Fractals in nature: from characterization to simulation. The Science of Fractal Images.

[CR55] Walker, E., Glover, P.W.J.: Permeability models of porous media: characteristic length scales, scaling constants and time-dependent electrokinetic coupling. Geophysics **75**, E235–E246 (2010)

[CR56] Yu B, Li J (2001). Some fractal characters of porous media. Fractals.

[CR57] Zhang, J.: Calculation of fractal dimension of a 3D volume using fft. MATLAB Central File Exchange. http://www.mathworks.com/matlabcentral/fileexchange/6964-calculation-of-fractal-dimension-of-a-3d-volume-using-fft (2005). Accessed 20 Jan 2015

[CR58] Zeybek, A.D., Onur, M.: Conditioning fractal (fBm/fGn) porosity and permeability fields to multiwell pressure data. Math. Geol. **35**, 577–612 (2003)

